# Aromatic systems with two and three pyridine-2,6-dicarbazolyl-3,5-dicarbonitrile fragments as electron-transporting organic semiconductors exhibiting long-lived emissions

**DOI:** 10.3762/bjoc.19.139

**Published:** 2023-12-12

**Authors:** Karolis Leitonas, Brigita Vigante, Dmytro Volyniuk, Audrius Bucinskas, Pavels Dimitrijevs, Sindija Lapcinska, Pavel Arsenyan, Juozas Vidas Grazulevicius

**Affiliations:** 1 Department of Polymer Chemistry and Technology, Kaunas University of Technology, Radvilenu pl. 19, LT-50254, Kaunas, Lithuania; 2 Latvian Institute of Organic Synthesis, Aizkraukles 21, LV-1006, Riga, Latvia

**Keywords:** charge transport, intramolecular charge transfer, photophysical properties, pyridine-3,5-dicarbonitrile

## Abstract

The pyridine-3,5-dicarbonitrile moiety has gained significant attention in the field of materials chemistry, particularly in the development of heavy-metal-free pure organic light-emitting diodes (OLEDs). Extensive research on organic compounds exhibiting thermally activated delayed fluorescence (TADF) has led to numerous patents and research articles. This study focuses on the synthesis and investigation of the semiconducting properties of polyaromatic π-systems containing two and three fragments of pyridine-2,6-dicarbazolyl-3,5-dicarbonitrile. The compounds are synthesized by Sonogashira coupling reactions and characterized by steady-state and time-resolved luminescence spectroscopy. The compounds show efficient intramolecular charge transfer (ICT) from the donor to the acceptor. The photoluminescence (PL) spectra of the solutions of the compounds showed non-structured emission peaks in the visible region, which are attributed to ICT emission. The PL intensities of the solutions of the compounds are enhanced after deoxygenation, which is indicative of TADF. The photoluminescence quantum yields and TADF properties of the compounds are sensitive to the medium. Cyclic voltammetry measurements indicate good hole-blocking and electron-injecting properties due to their high ionization potentials. Photoelectron spectroscopy and time-of-flight measurements reveal good electron-transporting properties for one of the compounds. In general, polyaromatic π-systems with pyridine-3,5-dicarbonitrile fragments demonstrate promising potential for use in organic electronic devices, such as OLEDs.

## Introduction

The pyridine-3,5-dicarbonitrile moiety attracted a great deal of attention in the last decade in the field of materials chemistry, precisely in the development of novel heavy-metal-free pure organic light-emitting diodes (OLEDs). Inexpensive and environmentally friendly emitters are vital for organic electronic devices including OLEDs. Thus, extensive search for organic dyes exhibiting E-type fluorescence (thermally activated delayed fluorescence (TADF)) is booming [1–3]. The majority of research results are protected by an impressive amount of patent applications.

**Figure 1:**
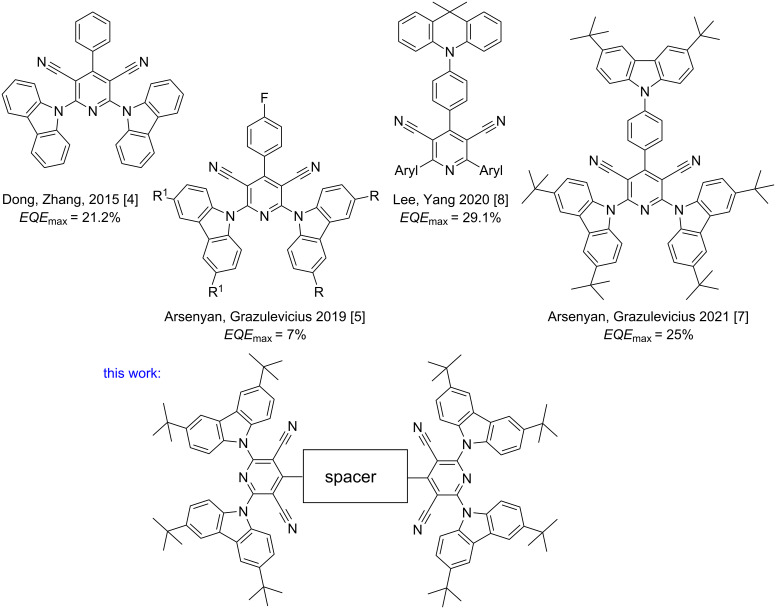
Chemical structures of pyridine-3,5-dicarbonitrile-based TADF emitters.

The first example of a blue TADF emitter based on a pyridine-3,5-dicarbonitrile scaffold (to serve as an electron acceptor) directly linked to a carbazole moiety (to serve as an electron donor) was reported by the groups of Dong and Zhang in 2015 [4]. 2,6-Di(9*H*-carbazol-9-yl)-4-phenylpyridine-3,5-dicarbonitrile (CPC) showed an extremely small singlet–triplet splitting and a fair photoluminescence quantum yield (PLQY). The optimized organic light-emitting diode (OLED) based on 13 wt % CPC doped in 1,3-bis(9*H*-carbazol-9-yl)benzene (mCP) as host exhibited maximum current efficiency, power efficiency, and external quantum efficiency (EQE) of 47.7 cd A^−1^, 42.8 lm W^−1^, and 21.2%, respectively (Figure 1). Since then, this moiety has been modified by many scientific groups to improve its electroactive properties.

Various pyridine-3,5-dicarbonitriles bearing substituted carbazoles were synthesized using different donating and accepting groups to achieve optimal charge-transporting and fluorescent properties within one TADF compound (Figure 1) [5]. The obtained TADF emitters (453 to 550 nm) show photoluminescence quantum yields of up to 98% in oxygen-free toluene solutions. These TADF emitters are suitable for OLEDs with brightness of 10 000 cd m^−2^, electroluminescence ranging from blue to yellow, maximum current of 15 cd/A and higher EQE than 7%.

Pyridine-3,5-dicarbonitrile-based TADF materials exhibit different visible light emission spectra (Figure 1). Recently, Chen and Lu reported two new orange-red/red TADF emitters composed of pyridine-3,5-dicarbonitrile-derived electron-acceptor and acridine electron–donor moieties. The films of molecular mixtures of these emitters with the hosts exhibited excellent photophysical properties [6]. They showed PLQY of up to 91%, tiny singlet–triplet energy gaps of 0.01 eV, and ultrashort TADF lifetimes of less than 1 µs. TADF-OLEDs based on these materials exhibited EQE_max_ of up to 25.0% and well-suppressed efficiency roll-offs. Green and orange normal/dual TADF emitters were produced using compounds containing 3,6-di-*tert*-butylcarbazole and 3,7-dibromophenothiazine moieties. Pyridine-3,5-dicarbonitriles substituted with 3,6-di-*tert*-butylcarbazole were successfully leveraged as TADF emitters in the fabrication of OLED with relatively high device life-times and a high EQE_max_ of 25% [7]. An effective green TADF was achieved for pyridine-3,5-dicarbonitriles with highly twisted conformations that contain dimethylacridan substituents [8]. OLEDs based on phenyl-substituted pyridine-3,5-dicarbonitrile showed very high EQE_max_ of 29.1% due to a high PLQY of 89% ascribed to the rigid acceptor geometry.

In addition to the utilization of pyridine-3,5-dicarbonitriles in OLEDs, an example of their application in photocatalysis has been recently published [9]. 2,6-Bis(4-cyanophenyl)-4-(9-phenyl-9*H*-carbazol-3-yl)pyridine-3,5-dicarbonitrile) exhibits two aggregate states in aqueous dispersions: amorphous nanospheres and ordered nanofibers with π–π molecular stacking. The nanofibers promoted the photocatalytic production of H_2_ while the nanospheres produced hydrogen peroxide (H_2_O_2_).

The introduction of the additional carbazolylphenyl moiety in the CPC molecule [4] allowed us to improve the EQE_max_ of an OLED to 25% [7]. In continuation of our studies in the field of the development of new TADF emitters [10], we would like to report our research results on the development of straightforward protocols for the synthesis and properties of polyaromatic π-systems with two and three pyridine-2,6-dicarbazolyl-3,5-dicarbonitrile fragments.

## Results and Discussion

### Synthesis

**Scheme 1:**
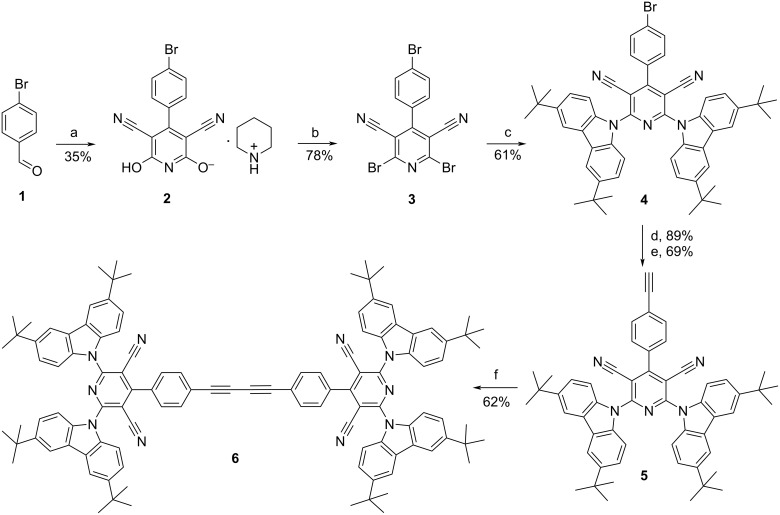
Synthesis of dicyanocarbazole **6**. Reaction conditions: a) cyanoacetamide, piperidine, methanol, 40 °C, 48 h; b) POBr_3_, 170 °C, 1 h; c) 3,6-di(*tert-*butyl)carbazole, NaH, THF, DMF, 0 → rt, 3 h; d) ethynyltrimethylsilane (2 equiv), PdCl_2_(PPh_3_)_2_ (0.07 equiv), CuI (0.05 equiv), diisopropylethylamine, DMF, 55 °C, 12 h; e) K_2_CO_3_ (2 equiv), MeOH/Et_2_O, 1 h; f) Pd(PPh_3_)_4_ (0.1 equiv), CuI (1 equiv), DMF/DIPEA, 80 °C, 48 h.

**Scheme 2:**
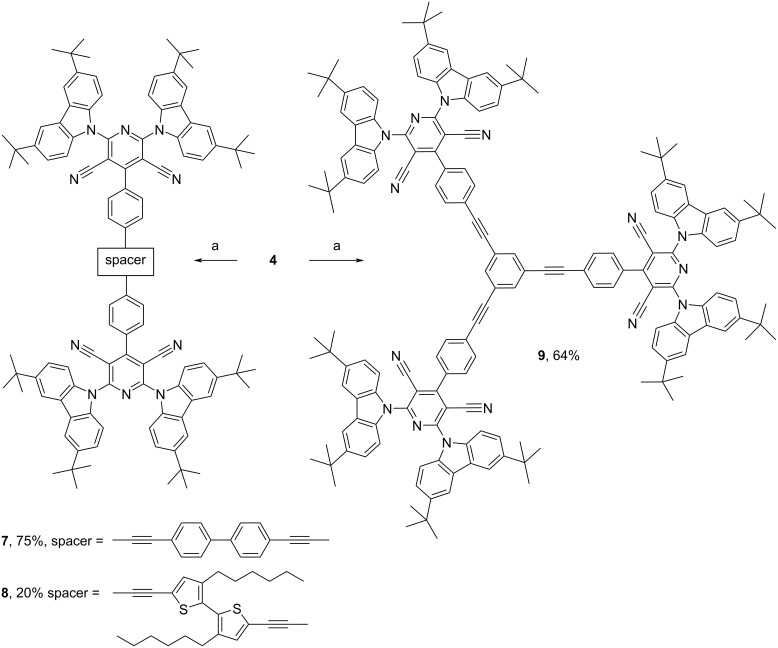
Synthesis of dicyanocarbazoles **7**–**9**. Reaction conditions: a) corresponding ethynyl arene, Pd(Ph_3_P)_4_ (5 mol %), Cu(I) (3 mol %), triethylamine, DMF, 90 °C, 18 h.

The sophisticated pyridine-3,5-carbonitriles **6**–**9** were synthesized starting with 4-bromobenzaldehyde (**1**) (Scheme 1 and Scheme 2). Thus, piperidinium 3,5-dicyano-6-hydroxy-4-(4-bromophenyl)pyridin-2-olate (**2**) was prepared by cyclocondensation of **1** with cyanoacetamide in methanol in the presence of piperidine. The conversion of **2** to the corresponding 2,6-dibromo-4-(4-bromophenyl)pyridine-3,5-carbonitrile (**3**) was carried out by melting of compound **2** with phosphorous oxybromide without any solvent at 170 °C for 1 h in good yield. According to the previously reported procedure [4–5], 2,6-bis(3,6-di-*tert*-butylcarbazol-9-yl)-4-(4-bromophenyl)pyridine-3,5-carbonitrile (**4**) was obtained by the interaction of 3,6-di-*tert*-butyl-9*H*-carbazole with compound **3** in THF/DMF solution. The ethynylphenyl-substituted pyridine **5** was synthesized by Sonogashira coupling of **4** with ethynyltrimethylsilane in the presence of PdCl_2_(PPh_3_)_2_ and copper(I) iodide in DMF/DIPEA solution at 55 °C with subsequent desilylation with potassium carbonate. Finally, butadiyne **6** was prepared by a homocoupling reaction of **5** with 80% yield. Derivatives containing two dicyanopyridyl moieties, **7** and **8**, were prepared starting with a Sonogashira coupling of compound **4** with 4,4’-diethynylbiphenyl and 5,5’-diethynyl-3,3’-dihexyl-2,2’-bithiophene, respectively. Finally, the snowflake-shaped compound **9** bearing three dicyanopyridyl moieties was constructed by the treatment of 1,3,5-triethynylbenzene with 3 equiv of **4**. The structural identity and purity of compounds **6**–**9** were confirmed by the spectral data and elemental analyses. Remarkably, despite a highly hindered structure, compound **9** exhibits good solubility in non-polar solvents.

### Photophysical properties

The electronic structures of compounds **6**–**9** in the ground and excited states were investigated by steady-state luminescence spectroscopy. Meanwhile, time-resolved spectroscopy at different temperatures was used to investigate the nature of long-lived emissions of **6**–**9** as it was previously done for the reference compound 2,6-bis(3,6-di-*tert*-butyl-9*H*-carbazol-9-yl)-4-(4-fluorophenyl)pyridine-3,5-carbonitrile (**REF**) [5].

**Figure 2:**
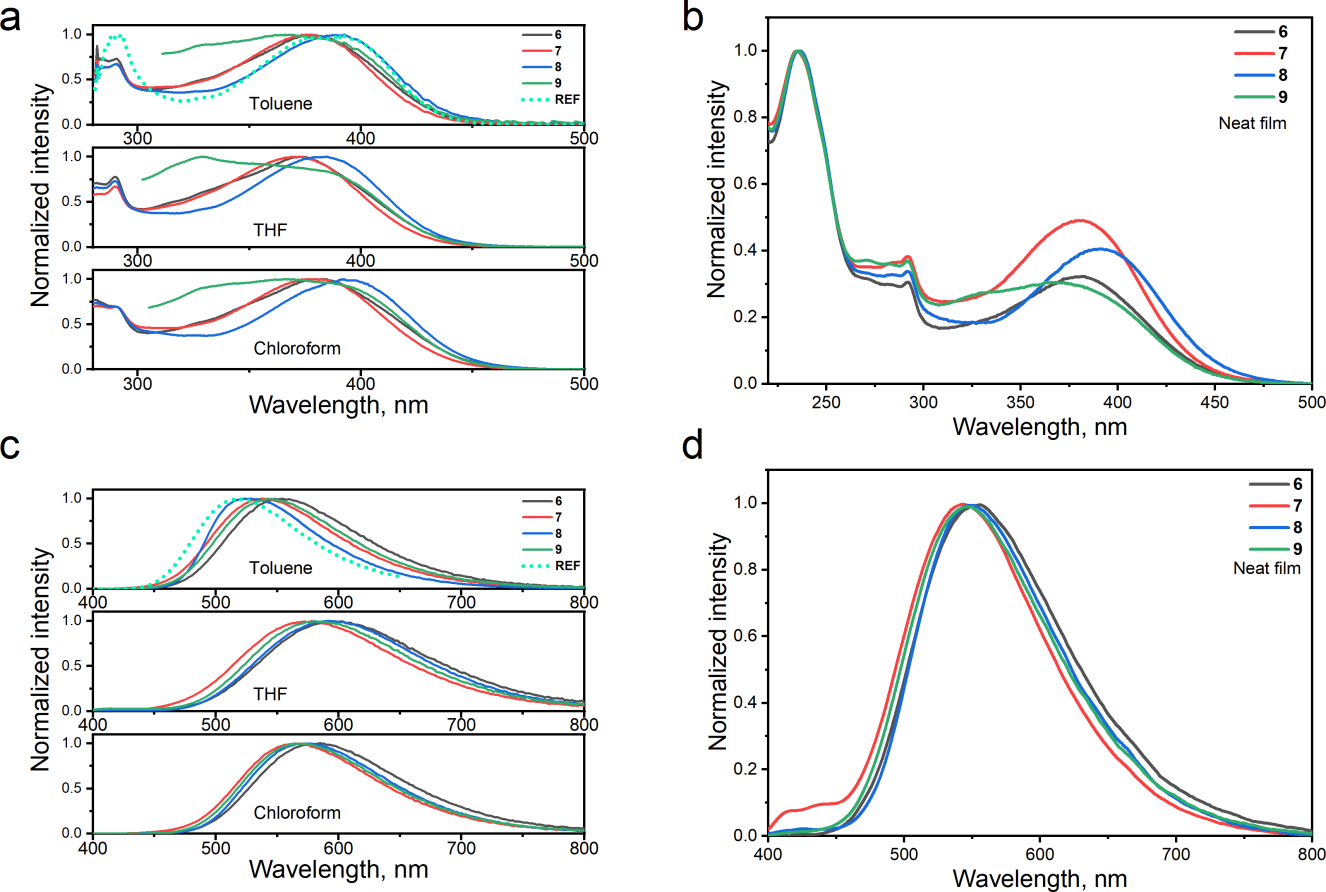
Absorption (a, b) and PL (c, d) spectra of dilute toluene, THF, and chloroform solutions (10^−5^ M) and of the films of compounds **6**–**9**.

Absorption spectra of dilute toluene, tetrahydrofuran (THF), and chloroform solutions as well as of the films of compounds **6**–**9** are shown in Figure 2a,b. The nonstructured low-energy bands at wavelengths of 350–450 nm are well seen in the absorption spectra of **6**–**9**. The wavelengths of peaks of these low-energy absorption bands are collected in Table 1. A similar band was previously observed for **REF** [5]. On the basis of the results of the theoretical investigations, the low-energy absorption band of **REF** has been attributed to the intramolecular charge transfer (ICT) caused by electron transfer from the donor to the acceptor. The absorption spectrum of **REF** is included in Figure 2a for comparison. Compounds **6**–**9** are characterized by similar absorption bands also caused by electron transfer from the 3,6-di-*tert*-butyl-9*H*-carbazole units to the pyridine-3,5-dicarbonitrile moiety. The number of **REF** fragments in the structures of compounds **6**–**9** does not have any strong effect on the positions of their absorption spectra. As a result, similar optical band gaps (*E*_g_^opt^*^.^*) of 2.74–2.8 eV were obtained for **6**–**9**. It should be noted that the 5,5’-diethynyl-3,3’-dihexyl-2,2’-bithiophene bridge of compound **8** had a slight impact on its red-shifted absorption spectra in comparison to that of other compounds (Figure 2a,b and Table 1).

**Table 1:** Common physical parameters of compounds **6**–**9**.

Compounds	Medium	**6**	**7**	**8**	**9**

λ_abs_, nm	toluene/film/mCP-based film	379/382/385	378/381/385	391/392/394	368/369/391
λ_PL_, nm		555/552/522	539/544/511	525/550/516	544/548/520
FWHM, nm		125/127/118	114/121/116	104/122/109	119/123/114
PLQY, %		6.7/4/6.33	7.6/6.4/7.3	17.1/7.4/9.6	9.15/6.3/8
Δ*E*_ST_, eV	mCP-based film	0.14	0.21	0.25	0.24
*E*_onset_,_ox_, V	CH_2_Cl_2_ solution	1.34	1.52	1.37	1.50
*E*_onset_,_ox vs Fc_, V		1.02	1.09	1.06	1.08
*E*_onset_,_red_, V		−1.15	−1.15	−1.32	−1.18
*E*_onset_,_red vs Fc_, V		−1.47	−1.58	−1.63	−1.60
*Fc*_onset_, V		0.32	0.43	0.31	0.42
*IP*_CV_, eV^a^		5.82	5.89	5.86	5.88
*EA*_CV_, eV^b^		3.33	3.22	3.17	3.20
*IP*^PE^, eV	film	6.15	6.21	6.20	6.04
*EA*^PE^, eV		3.37	3.41	3.46	3.28
µ_e_, cm^2^/V∙s		–	5.7 × 10^−6,c^	–	–
*T*_m_, °C^d^	powder	–	–	–	–
*T*_g_, °C^e^		63	75	80	90
*T*_d_, °C^f^		449	493	433	488
*M*, g/mol		1566	1718	1899	2424

^a^Calculated according to equation: *EA*_CV_ = e (*E*_ox 1/2 vs Fc_ − *Fc*_1/2_ + 4.8) [eV]; ^b^calculated according to equation: *IP*_CV_ = −e (*E*_red 1/2 vs Fc_ − *Fc*_1/2_ + 4.8) [eV]; ^c^at electric field of 8.1 × 10^5^ V/cm; ^d^melting temperature at the 1st/2nd heating scan with a rate of 10 °C/min, N_2_ atmosphere; ^e^glass-transition temperature at the 2nd heating scan; ^f^5% weight loss temperature at the scan rate of 20 °C/min, N_2_ atmosphere.

The photoluminescence spectra of toluene solutions of the compounds are characterized by non-structured shapes typical for ICT emissions (Figure 2c and Figure S1 in Supporting Information File 1) [11]. This interpretation is additionally supported by redshifted and broadened PL spectra of their THF and chloroform solutions with respect to the toluene solutions. For example, the intensity maxima at 525, 571, and 594 nm and full widths at half maxima (FWHM) of 105, 130, and 154 nm were obtained for toluene, chloroform, and THF solutions of compound **8**, respectively (Figure 2c, Table 1, and Figure S1 in Supporting Information File 1). In comparison to the PL spectrum of the toluene solution of **REF**, the PL spectra of toluene solutions of compounds **6**–**9** were redshifted. Since the freedom of movements (vibrations and rotations) of parts of **REF** are prevented in the solid state, the PL spectra of the films of compounds **6**–**9** were practically in the same spectral region (Figure 2d). It should be noted that the PL spectra of the neat films exhibit low-intensity high-energy bands and high-intensity low-energy bands. The presence of two distinct fluorescence bands is an indication of the existence of molecular conformations separated by a barrier. This is a characteristic of twisted internal charge transfer (TICT) states.

**Figure 3:**
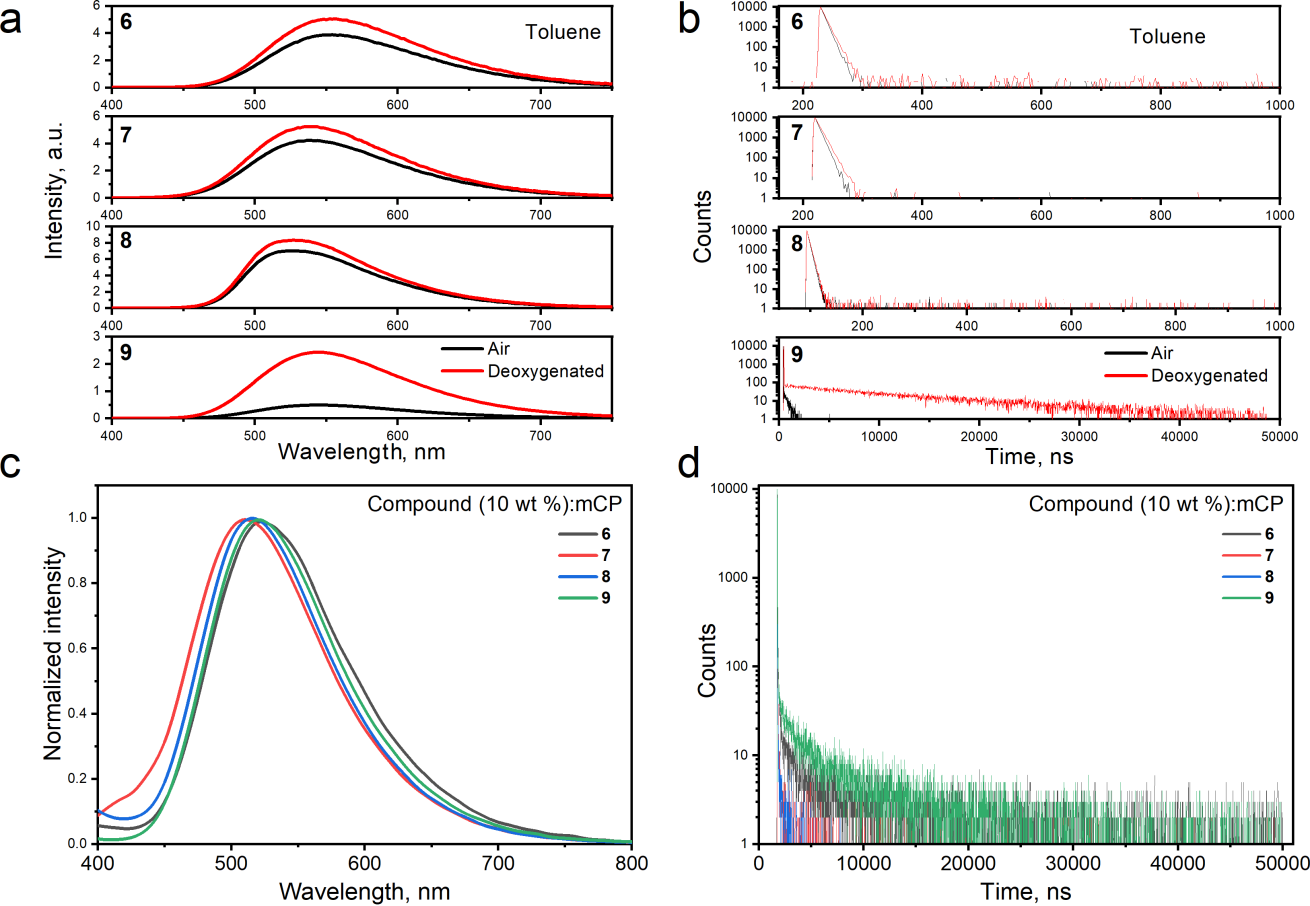
PL spectra (a) and PL decay curves (b) of air-equilibrated (as prepared) and deoxygenated toluene solutions of compounds **6**–**9**. PL spectra (c) and PL decay curves (d) of the films of molecular mixtures of mCP as host and compounds **6**–**9** (10 wt %).

It is worth of noting that the PL intensity of solutions of compounds **6**–**9** is increased after deoxygenation by argon (Figure 3a and Figure S2a in Supporting Information File 1). The increase of PL intensity is attributed to a delayed fluorescence turn-on when the triplet quenching by oxygen is omitted. This delayed fluorescence is TADF in nature as it is for compound **REF**. The increments of PL are different for the toluene, chloroform, and THF solutions. This observation shows that the TADF properties of compounds **6**–**9** are sensitive to the medium. For example, the ratios of PL intensities of deoxygenated and air-equilibrated toluene, chloroform, and THF solutions of 4.86, 7.24, and 1.41 were obtained for compound **9** (Figure 3a and Figure S2a, Supporting Information File 1). The TADF properties of derivatives **6**–**9** are discussed in more detail below. Long-lived emissions are detectable in the corresponding PL decay curves (Figure 3b and Figure S2b in Supporting Information File 1). The strongest PL intensity increment was detected for compound **9** containing the biggest number of dicyanopyridyl moieties. It can be concluded, that the TADF properties of **REF**-based molecules can be induced by linking additional **REF** moieties through the appropriate bridges. Similarly, the strongest TADF intensity was obtained for the neat films of compound **9** and of compound **9** molecularly dispersed in the host 1,3-bis(*N*-carbazolyl)benzene (mCP; Figure 3c,d and Figure S3a in Supporting Information File 1). Low-energy absorption bands of molecular mixtures of compounds **6–9** and mCP are attributed to ICT bands of **6**–**9** (see Figure S3b in Supporting Information File 1).

**Figure 4:**
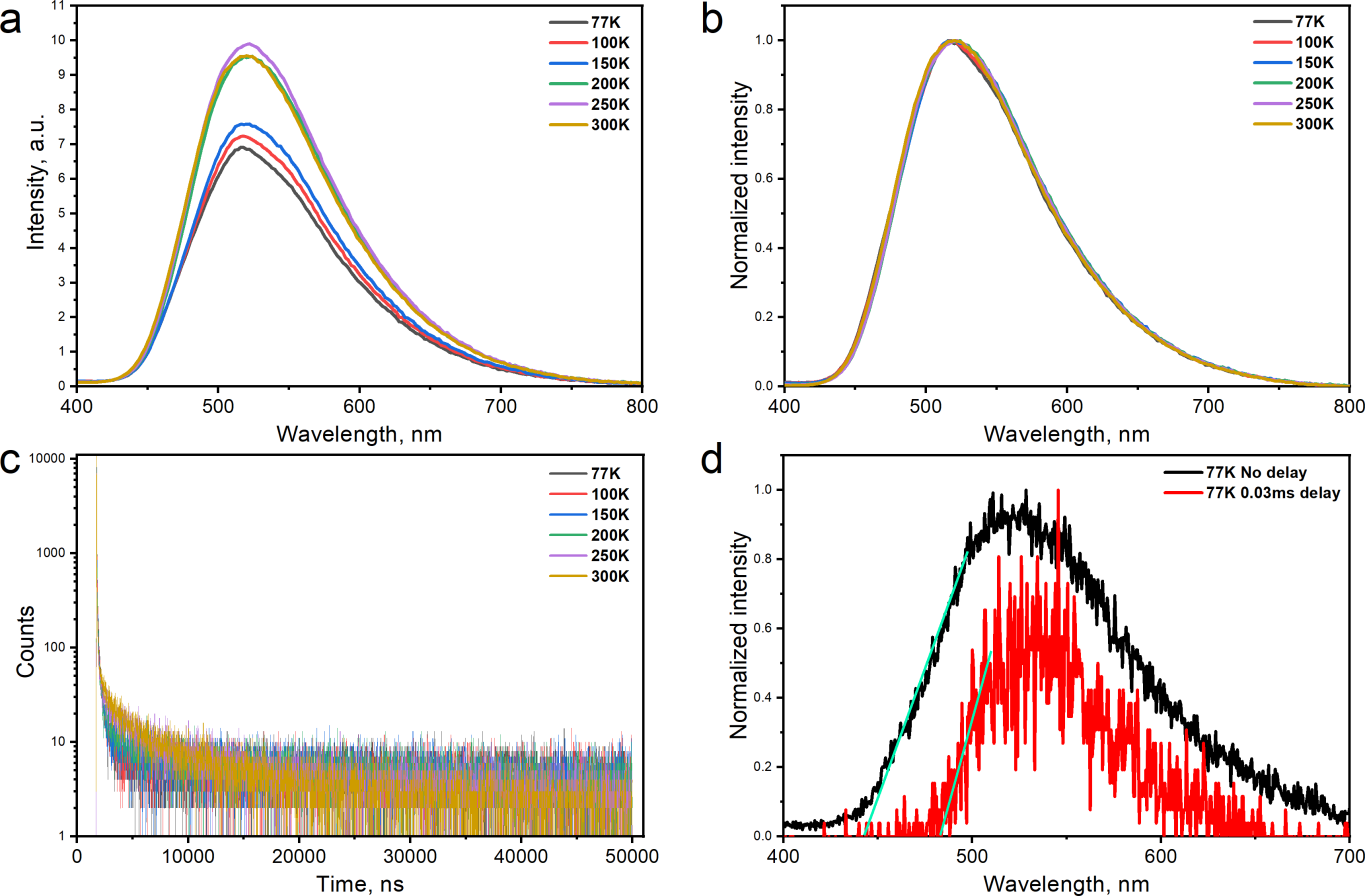
Non-normalized (a) and normalized (b) PL spectra and PL decay curves (c) of the film of a 10 wt % molecular dispersion of compound **9** in mCP recorded at the indicated different temperatures. PL and phosphorescence spectra of the same sample recorded at 77 K. (d) Phosphorescence was separated from fluorescence using a delay of 0.03 ms after excitation. Excitation wavelengths were 350 and 374 nm, respectively, for recording the PL spectra and PL decay curves.

The temperature dependencies of PL spectra and PL decay curves of the 10 wt % molecular dispersions of compounds **6**–**9** in mCP are plotted in Figure 4a,b and Figures S4 and S5 in Supporting Information File 1. The PL intensity increased with increasing temperature and the profiles of the PL spectra were practically the same at the different temperatures proving the fluorescence nature of long-lived emission (Figure 4c and Figures S4–S6 in Supporting Information File 1). Two decay regimes were obtained for the compounds **6**–**9**. The initial decay is attributed to prompt fluorescence and the other decay is related to delayed fluorescence. The intensity of delayed fluorescence grows with the temperature rising which is typical for TADF compounds [12]. Such processes are possible because of the relatively low energy gap (Δ*E*_ST_) between the lowest singlet and triplet states (Figure 4d and Figure S7 in Supporting Information File 1). A Δ*E*_ST_ value of 0.24 eV was obtained for compound **9**. The highest photoluminescence quantum yield (PLQY) of 17.1% was obtained for the dilute toluene solution of compound **8** (Table 1). The neat film of compound **8** showed a more than twice lower PLQY which was enhanced using the mCP host. The other compounds showed similar PLQY trends (Table 1).

### Thermal properties

**Figure 5:**
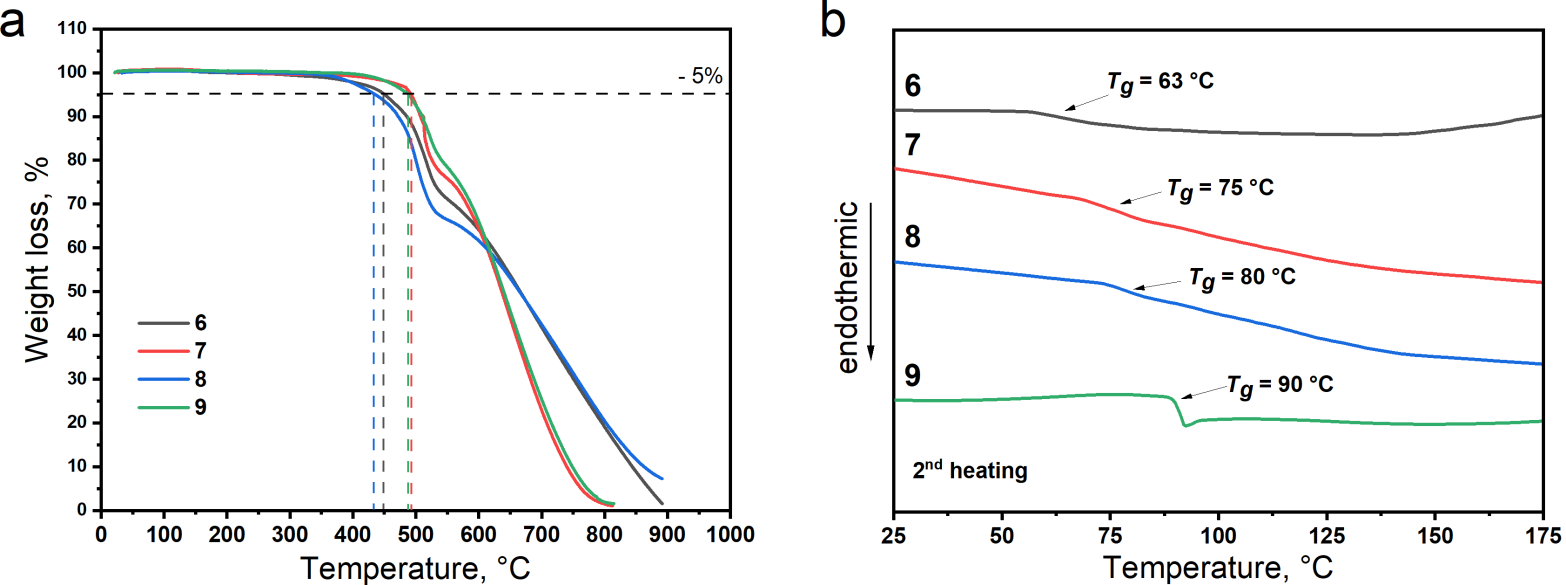
TGA (a) and DSC 2nd heating (b) curves of compounds **6**–**9**.

The thermal stability and phase transitions of the synthesized derivatives **6**–**9** were investigated utilizing thermogravimetric analysis (TGA) and differential scanning calorimetry (DSC), respectively. The experimental data are shown in Figure 5 and the characteristics are collected in Table 1.

An amorphous character of the derivatives was identified. No endothermic (melting) or exothermic (crystallization) transitions were observed during the 1st and 2nd DSC heating and cooling scans. However, glass transitions were detected for all the studied compounds. The glass transition temperatures (*T*_g_) fall in the range between 63 and 93 °C (Figure 5b). A correlation between the glass transition temperature and the molecular weight of compounds **6**–**9** was observed. The *T*_g_ values of the derivatives increase gradually by 5–12 °C starting from compound **6** which possesses the lowest molecular weight (*M =* 1566 g/mol) up to compound **9** which possesses the highest *M* value (2424 g/mol) [13]. A high thermal stability was observed for all derivatives **6**–**9**. Their 5% weight loss temperatures (*T*_d_) ranged from 433 to 493 °C (Figure 5a and Table 1).

### Electrochemical properties

**Figure 6:**
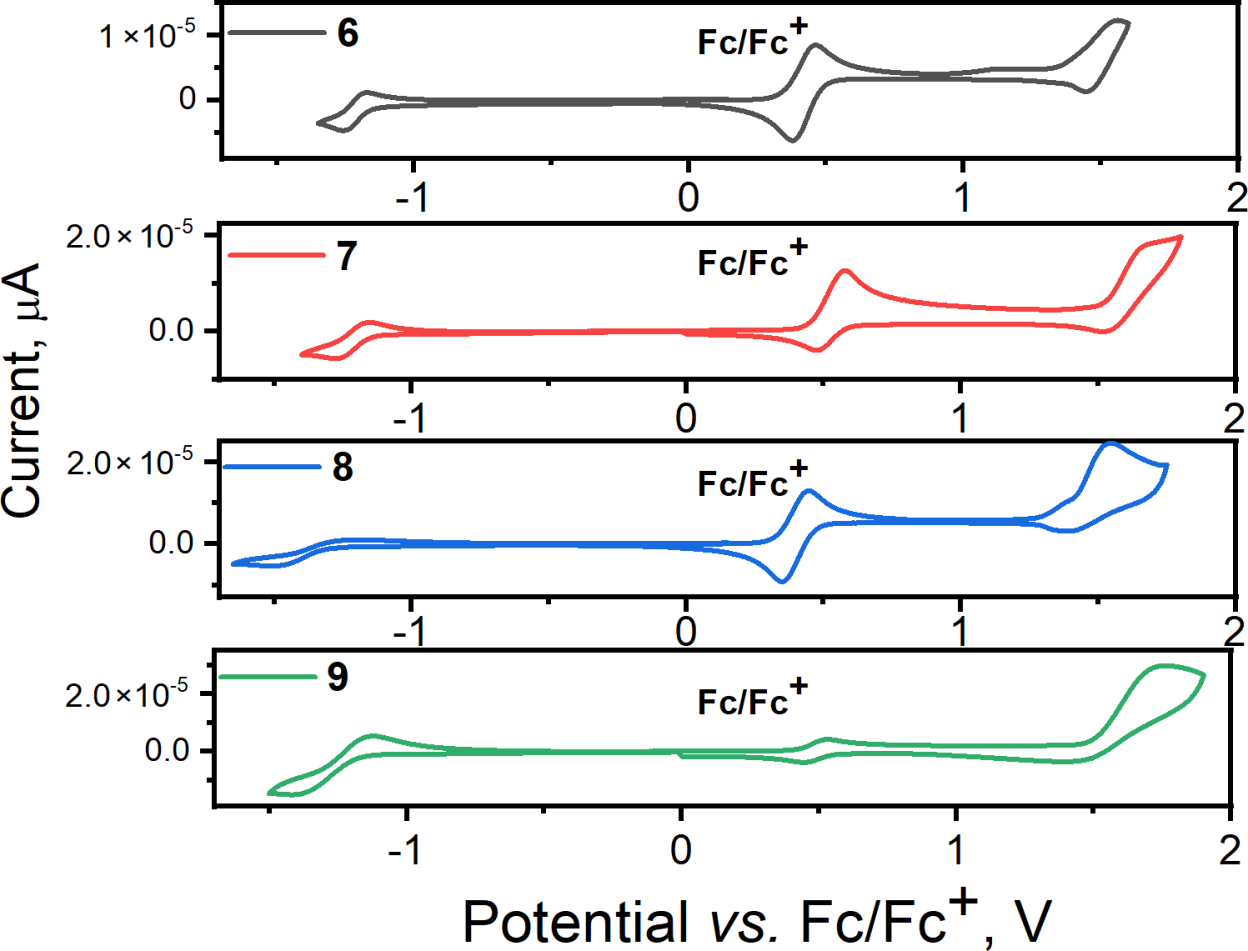
CV curves of compounds **6**–**9**.

The electrochemical properties of compounds **6**–**9** were studied by cyclic voltammetry (CV) selecting the electrochemical window from −1.6 to 1.7 V and the CV data were corrected using ferrocene (*Fc*) as inner standard. The CV curves are shown in Figure 6 and the data are collected in Table 1. The reduction onset potentials (*E*_red,onset_) of compounds **6**–**9** fall in the range from −1.63 to −1.47 V, while the onset values of the first oxidation wave range from 1.02 to 1.09 V. The values of *E*_red,onset_ are comparable with those of recently published compounds containing a dicyanopyridyl moiety as a central electron-accepting core [7]. In case of oxidation potentials (0.96 to 1.04 V) there is only a slight effect of the extended conjugation between the dicyanopyridyl moieties. The results of CV measurements were used to obtain the ionization potential (*IP*_CV_) and electron affinity (*EA*_CV_) values of the compounds **6**–**9**. These values were obtained using the following equations: *IP*_CV_ = e (*E*_onset,red_ − *Fc*_onset_ + 4.8), *EA*_CV_ = e (*E*_onset,ox_ − *Fc*_onset_ + 4.8) [7]. The *IP*_CV_ values fall in a range of 5.80–5.90 eV, while the electron affinities (*EA*_CV_) vary from 3.20 to 3.33 eV (Table 1). The obtained values are comparable to the corresponding ionization potentials (*IP*_PE_) and electron affinities (*EA*_PE_) obtained for the films by photoelectron emission spectrometry, presented and discussed in the following section. Small differences between the results obtained for the solutions and films appear to be due to different intermolecular interactions, which differ in solid films (between target molecules only) and solutions (between target molecules and molecules of the solvent) of the investigated compounds [14–15]. Compared to the results previously reported for compounds possessing a single dicyanopyridyl moiety [7–8,16], the studied compounds showed higher *IP* and *EA* values. This observation can be explained by the presence of additional dicyanopyridyl fragments in compounds **6**–**9**.

### Photoelectrical and charge-transporting properties

**Figure 7:**
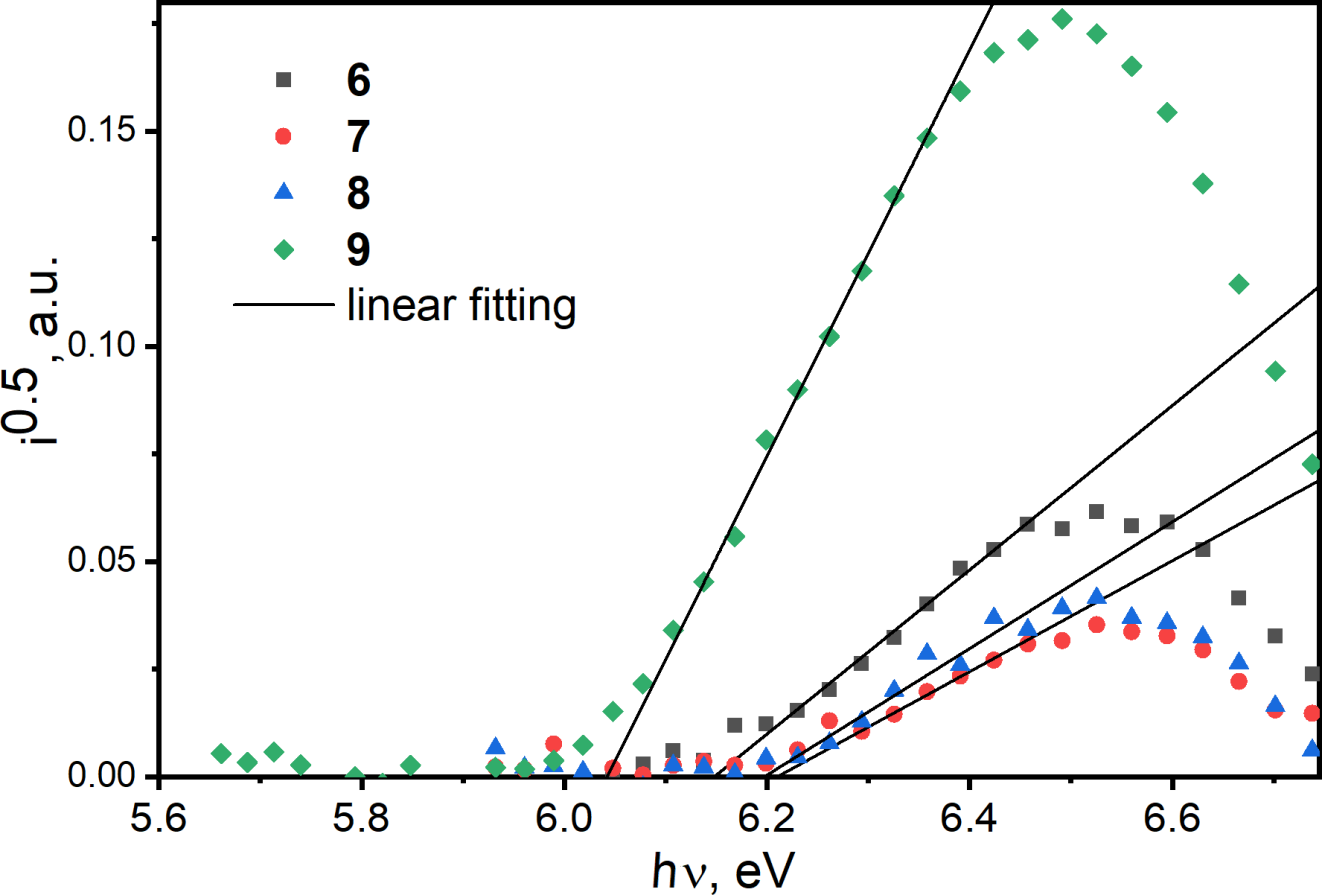
Photoelectron emission spectra of the vacuum-deposited films of compounds **6**–**9** on glass substrates covered by FTO electrodes.

The ionization potentials (*IP*^PE^) of thin films were obtained by photoelectron emission (PE) spectroscopy in air (Figure 7 and Table 1). To generate photoelectrons detectable by a counter electrode within the PE experiments, a low-power deuterium UV lamp was used. It was previously shown that the PE technique gives practically the same *IP*^PE^ values as they are recorded by other methods in vacuum [17]. The PE spectra as current (*i*) versus photon energy (*h*ν) dependences for thin films of compounds **6**–**9** are built in the square root of *i* versus linear *h*ν plots according to the relation *i* ~ (*h*ν *− IP*^PE^)^2^ [18]. The ionization potentials, *IP*^PE^, were obtained by linear fitting of the linear parts of the plots [19], and the *IP*^PE^ values were taken at crossing points of the fitting curves with the baseline (*x*-axis). Compounds **6**–**9** are characterized by rather good hole-blocking properties due to the high *IP*^PE^ values of 6.04–6.21 eV. In contrast, they are characterized by pertinent electron-injecting properties because of the electron affinities (*EA*^PE^) of 3.28–3.46 eV. The *EA*^PE^ values were calculated by the equation *EA*^PE^ = *IP*^PE^ − *E_g_*^opt^*^.^* using *E*_g_^opt.^ taken from the low-energy edge of the absorption spectra of the corresponding films (Figure 2b, Table 1).

**Figure 8:**
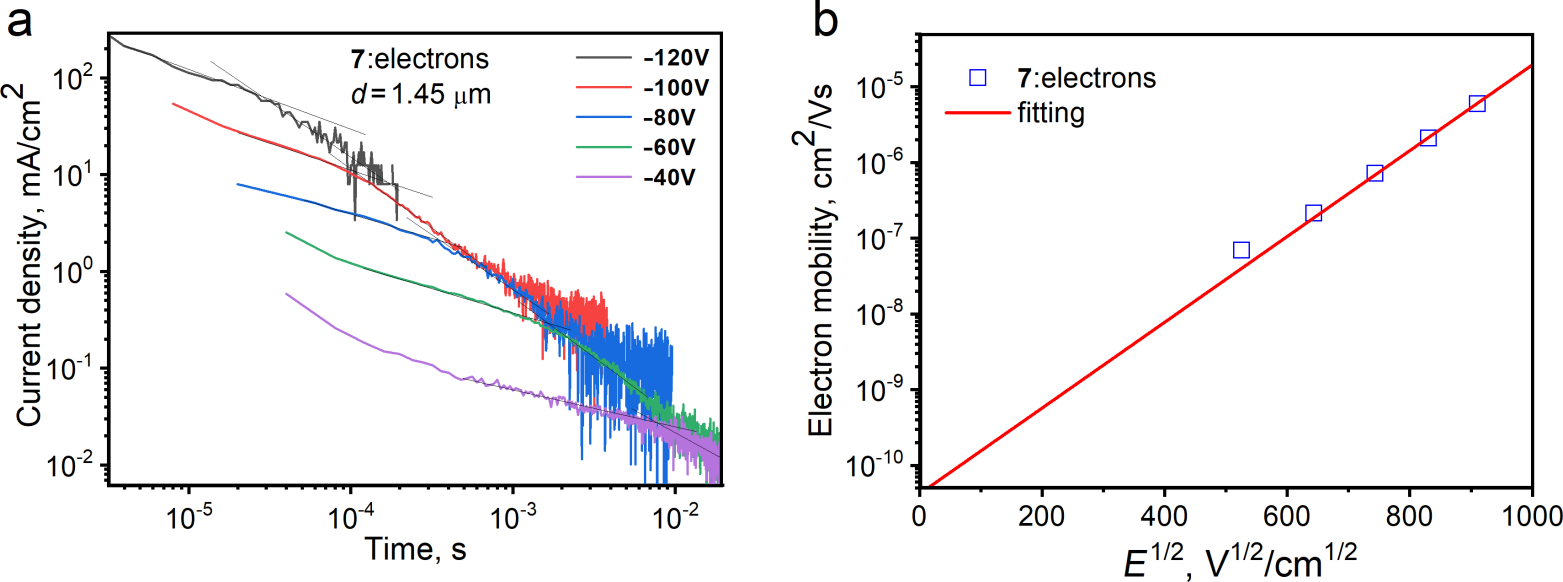
The current transients (a) for electrons recorded at the different voltages for the vacuum-deposited films of compound **7** in a diode-like structure ITO/film/Al. Excitation wavelength 355 nm, laser pulse duration 6 ns. b) Electric field dependence of electron mobility for the layer of **7** at room temperature (295 K).

We used time of flight (TOF) measurements to investigate the charge-transporting properties of compounds **6**–**9**. According to the current transients for electrons with clearly seen transit times in log–log scales, compound **7** is characterized by electron transport. The recorded current transients for electrons in vacuum-deposited films of compound **7** show moderate dispersion (Figure 8a). In contrast, the hole transport was not detected, apparently because of the low mobility of holes being out of the detectable range of TOF measurements. The similar situation was in the case of the other compounds which showed current transients without detectable transit times (Figure S7 in Supporting Information File 1). The electron mobility (µ_e_) as the function of the electric field was substantial for **7** with the field dependence parameter (β) of 0.013 V/cm. This parameter was obtained by fitting according to the formula of Poole–Frenkel-type mobility (
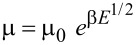
). Compound **7** is characterized by both good electron injecting and electron-transporting properties due to its *EA*^PE^ of 3.45 eV and electron mobility reaching 5.7 × 10^−6^ cm^2^/V∙s at the electric field of 8.1 × 10^5^ V/cm (Figure 8).

## Conclusion

Synthetically convenient protocols for the preparation of sophisticated pyridine-3,5-carbonitriles containing carbazole substituents in positions 2 and 6 were developed starting with 4-bromobenzaldehyde. The compounds were found to be soluble in common organic solvents and to exhibit non-structured emission peaks in the green-yellow color region of the spectrum. The PL intensity of the compounds in solution was enhanced after deoxygenation, indicating the presence of triplet harvesting by the mechanism of thermally activated delayed fluorescence. The optical band gaps of the compounds were determined to be 2.74–2.8 eV, while cyclic voltammetry measurements demonstrated that the compounds possess excellent hole-blocking properties due to their high ionization potentials. Moreover, they exhibited appropriate electron-injecting properties, as evidenced by their electron affinities. Photoelectron emission spectroscopy results confirmed the favorable electron-transporting properties with high ionization potentials, supporting their potential application as electron-transporting materials in OLEDs.

Overall, the polyaromatic π-systems with pyridine-3,5-dicarbonitrile fragments demonstrated a range of attractive properties, including efficient TADF, favorable charge transport, and thermal stability. These findings open up new possibilities for the design and development of high-performance OLEDs and other organic electronic devices. Further investigations and optimizations of the properties of such compounds may lead to the realization of environmentally friendly and cost-effective OLED technologies.

## Experimental

### Synthesis

Unless otherwise stated, all reagents were purchased from commercial suppliers and used without further purification. Thin-layer chromatography (TLC) was performed using Merck Silica gel 60 F254 plates and visualized by UV (254 nm) fluorescence. Zeochem silica gel (ZEOprep 60/35–70 microns – SI23501) was used for column chromatography. ^1^H and ^13^C NMR spectra were recorded on a Bruker 400 spectrometer at 400 and 101 MHz, respectively, at 298 K in CDCl_3_. The corresponding spectra are given in Supporting Information File 1. The ^1^H chemical shifts are given relative to residual CHCl_3_ signal (7.26 ppm), ^13^C chemical shifts are given relative to CDCl_3_ (77.16 ppm). The melting points were determined on a “Digital melting point analyzer” (Fisher), and the results are given without correction. Reagents and solvents were purchased from common vendors such as ACROS, Fluorochem, ABCR, and others.

**Piperidinium 3,5-dicyano-6-hydroxy-4-(4-bromophenyl)pyridin-2-olate** (**2**)**.** A mixture of cyanoacetamide (10.51 g, 0.125 mol, 2.5 equiv), piperidine (14.81 mL, 0.15 mol, 3 equiv), and MeOH (150 mL) was heated until complete dissolution. The mixture was cooled to rt and 4-bromobenzaldehyde (**1**, 9.25 g, 0.05 mol, 1 equiv) was added. The mixture was vigorously stirred at 40 °C for 48 h. Then solvents were evaporated to a half of volume and after cooling the precipitate was filtered. The crude product was washed with cold MeOH and EtOAc and dried under reduced pressure yielding **2** (7.40 g, 35%) as white powder, which was used in the next reaction without further purification. ^1^H NMR (400 MHz, DMSO-*d*_6_) 7.67–7.66 (m, 2H), 7.37–7.33 (m, 2H), 3.02–2.99 (m, 4H), 1.63–1.51 (m, 6H); MS–ESI^−^ (*m/z*): 315 ([M − H − piperidine]^−^, 100).

**2,6-Dibromo-4-(4-bromophenyl)pyridine-3,5-dicarbonitrile (3)**. A mixture of compound **2** (1.16 g, 2.89 mmol, 1 equiv) and POBr_3_ (2.49 g, 8.68 mmol, 3 equiv) was heated with stirring in an oil bath at 170 °C for 1 h. After cooling, the reaction was quenched by the addition of ice and the precipitate was filtered off. The crude product was purified by flash chromatography on silica gel using chloroform as eluent yielding compound **3** (1.0 g, 78%) as white powder. Mp > 200 °C; ^1^H NMR (400 MHz, DMSO-*d*_6_) 7.91–7.79 (m, 2H), 7.63–7.50 (m, 2H); GC–MS: 442 [M]^+^.

**2,6-Bis(3,6-di-*tert*-butylcarbazol-9-yl)-4-(4-bromophenyl)pyridine-3,5-dicarbonitrile** (**4**). Sodium hydride (60% oil dispersion, 180 mg, 5.36 mmol, 3.2 equiv) was added to THF (15 mL) under Ar atmosphere. Then, 3,6-di-*tert*-butyl-9*H*-carbazole (1.40 g, 5.3 mmol, 3 equiv) was added at 0 °C, the suspension was stirred for 30 min and then, a DMF (5 mL) solution of compound **3** (0.74 g, 1.67 mmol, 1 equiv) was added. The mixture was stirred at rt for 3 h (TLC) before it was poured into ice-water. The suspension was extracted with chloroform (5 × 30 mL) and dried over Na_2_SO_4_. After the solvent was removed, the crude yellow product was carefully washed twice with acetonitrile to remove the starting materials. Further purification was achieved using flash chromatography on silica gel (eluent: dichloromethane/petroleum ether 1:2). Compound **4** (0.86 g, 61%) was obtained as bright yellow powder. Mp > 200 °C; ^1^H NMR (400 MHz, CDCl_3_) 8.10 (d, *J* = 1.6 Hz, 4H), 7.86 (d, *J* = 8.5 Hz, 2H), 7.76 (d, *J* = 8.5 Hz, 2H), 7.71 (d, *J* = 8.7 Hz, 4H), 7.48 (dd, *J* = 7.8, 1.9 Hz, 4H), 1.46 (s, 36H); MS–ESI^+^ (*m/z*): 839 ([M + H]^+^, 100); Anal. calcd for C_53_H_52_BrN_5_: C, 75.88; H, 6.25; N, 8.35; found: C, 75.56; H, 6.30; N, 8.45.

**2,6-Bis(3,6-di-*tert*-butylcarbazol-9-yl)-4-(4-ethynylphenyl)pyridine-3,5-dicarbonitrile (5)**. A mixture of compound **4** (1.0 g, 1.19 mmol, 1 equiv), PdCl_2_(PPh_3_)_2_ (58 mg, 0.083 mmol, 0.07 equiv), CuI (11 mg, 0.06 mmol, 0.05 equiv) in 2 mL of DMF and 2 mL of DIPEA was degassed with Ar. Then, ethynyltrimethylsilane (0.34 mL, 2.38 mmol, 2 equiv) was added and the reaction mixture was stirred at 55 °C overnight. The mixture was poured into sat. aqueous solution of NH_4_Cl and extracted with dichloromethane (4 × 60 mL). The combined extracts were washed with water, brine, and dried over Na_2_SO_4_. After evaporation of the solvent, the crude residue was purified by flash chromatography on silica gel using a mixture of chloroform/petroleum ether/acetone 9:12:0.4 as eluent. 2,6-Bis(3,6-di-*tert*-butyl-9*H*-carbazol-9-yl)-4-(4-(trimethylsilylethynyl)phenyl)pyridine-3,5-dicarbonitrile (0.90 g, 89%) was obtained as bright yellow powder. Mp > 200 °C; ^1^H NMR (400 MHz, CDCl_3_) 8.11 (d, *J* = 1.9 Hz, 4H), 7.87–7.76 (m, 4H), 7.72 (d, *J* = 8.9 Hz, 4H), 7.49 (dd, *J* = 8.8, 1.9 Hz, 4H), 1.47 (s, 36H), 0.31 (d, *J* = 1.0 Hz, 9H); ^13^C NMR (101 MHz, CDCl_3_) 163.16, 154.51, 146.43, 136.98, 132.85, 132.58, 129.64, 127.20, 125.71, 124.18, 116.57, 114.00, 112.34, 103.64, 99.21, 98.30, 34.90, 31.81; MS–ESI^+^ (*m/z*): 856 ([M + H]^+^, 100). Next, potassium carbonate (69 mg, 0.5 mmol, 1 equiv) was added to a solution of 2,6-bis-(3,6-di-*tert*-butyl-9*H*-carbazol-9-yl)-4-(4-(trimethylsilylethynyl)phenyl)pyridine-3,5-dicarbonitrile (430 mg, 0.5 mmol, 1 equiv) in methanol (70 mL) and diethyl ether (20 mL) and the mixture was stirred under Ar for 1 h (TLC control). Then, the reaction mixture was diluted with dichloromethane and poured into water. The aqueous layer was extracted with dichloromethane (2 × 50 mL), the combined organic extracts were washed with water and brine, and dried over Na_2_SO_4_. After removal of the solvent the residue was purified by flash chromatography on silica gel, eluting with DCM/petroleum ether 1:1.5 → 1:1. Compound **5** was obtained (270 mg, 69%) as bright yellow powder. Mp > 200 °C; ^1^H NMR (400 MHz, CDCl_3_) 8.10 (dd, *J* = 2.0, 0.6 Hz, 4H), 7.89–7.78 (m, 4H), 7.71 (dd, *J* = 8.7, 0.6 Hz, 4H), 7.48 (dd, *J* = 8.8, 2.0 Hz, 4H), 3.28 (s, 1H), 1.46 (s, 36H).

**4,4'-(Buta-1,3-diyne-1,4-diylbis(4,1-phenylene))bis(2,6-bis(3,6-di-*tert*-butyl-9*H*-carbazol-9-yl)pyridine-3,5-dicarbonitrile) (6)**. A solution of compound **5** (130 mg, 0.166 mmol, 1 equiv) in DMF (2 mL) and DIPEA (1 mL) was bubbled with Ar. Then, Pd(PPh_3_)_4_ (19 mg, 0.0166 mmol, 0.1 equiv) and CuI (32 mg, 0.166 mmol, 1 equiv) were added and the mixture was stirred at 80 °C for 48 h. The reaction mixture was diluted with water and the product was extracted with dichloromethane (3 × 40 mL), washed with water, and brine. After evaporation of solvents the crude product was purified by flash chromatography on silica gel with DCM/petroleum ether 1:2 → 1:1. Derivative **6** was obtained as yellow powder (80 mg, 62%). Mp > 200 °C; IR ν_max_ (film): 2227, 1550; ^1^H NMR (400 MHz, CDCl_3_) 8.11 (dd, *J* = 2.0, 0.6 Hz, 8H), 7.90 (s, 8H), 7.73 (dd, *J* = 8.8, 0.5 Hz, 8H), 7.49 (dd, *J =* 8.8, 2.0 Hz, 8H), 1.46 (s, 72H); ^13^C NMR (101 MHz, CDCl_3_) 162.83, 154.53, 154.50, 146.56, 146.52, 136.96, 133.59, 133.50, 133.11, 129.93, 129.72, 126.17, 125.78, 125.76, 125.54, 124.23, 124.21, 116.61, 114.01, 112.43, 99.12, 99.00, 34.93, 31.83; Anal. calcd for C_110_H_104_N_10_: C, 84.36; H, 6.69; N, 8.94; found: C, 83.97; H, 6.70; N, 8.70.

### General method for the synthesis of compounds **7**–**9**

A mixture of 2,6-bis(3,6-di-*tert*-butyl-9*H*-carbazol-9-yl)-4-(4-bromophenyl)pyridine-3,5-dicarbonitrile (**4**, 168 mg, 0.2 mmol, 1 equiv), tetrakis(triphenylphosphine)palladium(0) (11.6 mg, 0.01 mmol, 0.05 equiv), CuI (1.2 mg, 0.006 mmol, 0.03 equiv) in 2 mL of DMF was degassed with Ar. Then, the appropriate ethynyl derivative (0.24 mmol, 1.2 equiv) and 1 mL of triethylamine were added and the resulting mixture was heated at 90 °C for 18 h. Then, the mixture was poured on ice and extracted with dichloromethane (4 × 30 mL). The solution was filtered through celite and dried over Na_2_SO_4_. The solvent was removed under reduced pressure and the yellow powder was purified by flash chromatography on silica gel.

**4,4’-(([1,1’-Biphenyl]-4,4’-diylbis(ethyne-2,1-diyl)bis(4,1-phenylene))bis(2,6-bis(3,6-di-*tert*-butyl-9*H*-carbazol-9-yl)pyridine-3,5-dicarbonitrile) (7)**. Two equiv of compound **4**, 1 equiv of 4,4’-diethynylbiphenyl were employed. After evaporation of the solvent the crude product was purified by flash chromatography on silica gel with dichloromethane/petroleum ether 1:1 → 2:1. Compound **7** was isolated as yellow powder (193 mg, 75%). Mp > 200 °C; IR ν_max_ (film): 2228, 1602, 1538, 1530; ^1^H NMR (400 MHz, CDCl_3_) 8.12 (d, *J* = 2.0 Hz, 8H), 7.95–7.83 (m, 8H), 7.74 (d, *J* = 8.8 Hz, 8H), 7.72–7.64 (m, 8H), 7.50 (dd, *J* = 8.8, 2.0 Hz, 8H), 1.47 (s, 72H); ^13^C NMR (101 MHz, CDCl_3_) 163.18, 154.58, 146.47, 140.57, 137.01, 132.54, 132.44, 132.41, 129.87, 127.40, 127.07, 125.76, 124.22, 122.07, 116.61, 114.14, 112.40, 99.16, 34.93, 31.84; Anal. calcd for C_122_H_112_N_10_: C, 85.28; H, 6.57; N, 8.15; found: C, 84.98; H, 6.60; N, 8.00.

**4,4’-(((3,3’-Dihexyl-[2,2’-bithiophene]-5,5’-diyl)bis(ethyne-2,1-diyl)bis(4,1-phenylene)bis(2,6-bis(3,6-di-*tert*-butyl-9*H*-carbazol-9-yl)pyridine-3,5-dicarbonitrile) (8)**. Two equiv of **4** per 1 equiv of 5,5’-diethynyl-3,3’-dihexyl-[2,2’]bithiophene were employed. After evaporation of the solvent the crude product was purified by flash chromatography on silica gel with chloroform/petroleum ether/acetone 9:15:0.4 by volume. The product **8** was obtained as orange powder (172 mg, 20%). Mp > 200 °C; IR ν_max_ (film): 2252, 2240, 1538, 1532, 1425; ^1^H NMR (400 MHz, CDCl_3_) 8.11 (d, *J* = 2.0 Hz, 8H), 7.93–7.80 (m, 8H), 7.73 (d, *J* = 8.7 Hz, 8H), 7.50 (dd, *J* = 8.8, 2.0 Hz, 8H), 7.27 (s, 2H), 2.56 (t, *J* = 7.8 Hz, 4H), 1.62–1.56 (m, 4H), 1.46 (s, 72H), 1.36–1.29 (m, 12H), 0.94–0.84 (m, 6H); ^13^C NMR (101 MHz, CDCl_3_) 163.10, 154.57, 146.47, 143.22, 137.00, 134.50, 132.46, 132.24, 130.63, 129.89, 127.06, 125.74,124.21, 122.56, 116.60, 114.11, 112.38, 99.13, 92.84, 86.01, 34.93, 31.83, 31.65, 30.67, 29.07, 28.89, 22.60, 14.12; Anal. calcd for C_130_H_132_N_10_S_2_: C, 82.24; H, 7.01; N, 7.38; found: C, 81.59; H, 7.13; N, 6.95.

**4,4’,4’’-((Benzene-1,3,5-triyltris(ethyne-2,1-diyl)tris(benzene-4,1-diyl))tris(2,6-bis(3,6-di-*tert*-butyl-9*H*-carbazol-9-yl)pyridine-3,5-dicarbonitrile) (9)**. Three equiv of compound **4** per 1 equiv of 1,3,5-triethynylbenzene were employed. After evaporation of the solvent the crude product was purified by flash chromatography on silica gel eluting with DCM/petroleum ether 1:2 → 2:1. The product **9** was obtained as orange powder (280 mg, 64%). Mp > 200 °C; IR ν_max_ (film): 2229, 1608, 1532; ^1^H NMR (400 MHz, CDCl_3_) 8.13–8.08 (m, 12H), 7.96–7.87 (m, 12H), 7.82 (s, 3H), 7.76–7.71 (m, 12H), 7.50 (dd, *J* = 8.8, 2.0 Hz, 12H), 1.46 (s, 108H); ^13^C NMR (101 MHz, CDCl_3_) 163.09, 154.56, 146.50, 137.00, 132.86, 132.69, 129.93, 126.80, 125.76, 124.23, 116.61, 114.09, 112.40, 99.15, 90.69, 34.93, 31.83; Anal. calcd for C_171_H_159_N_15_: C, 84.72; H, 6.61; N, 8.67; found: C, 84.54; H, 6.60; N, 8.55.

### Measurements

In this experimental investigation, thermogravimetric analysis (TGA) and differential scanning calorimetry (DSC) were employed to characterize the thermal properties of the samples. The measurements were performed using a TA instrument TGA Q50 apparatus for TGA and a TA instrument DSC Q2000 series thermal analyzer for DSC. During the TGA experiments, the samples were subjected to controlled heating at a rate of 20 °C/min under a nitrogen atmosphere. TGA allows us to observe the weight changes of the samples as a function of temperature, providing valuable insights into processes such as decomposition, volatilization, and oxidation. In the case of DSC measurements, the samples were heated at a rate of 10 °C/min in a nitrogen environment. DSC allows us to investigate the enthalpy changes associated with phase transitions, crystallization, and other thermal events occurring in the samples. The choice of a nitrogen atmosphere in both TGA and DSC experiments is significant as it helps to prevent undesired reactions with atmospheric components, ensuring a controlled and inert environment. These optimized experimental conditions enable accurate and reproducible data acquisition.

Ionization potential (*IP*^PE^) determination: The *IP*^PE^ analysis was conducted using electron photoemission spectrometry, a technique for investigating the energy required to remove an electron from a solid material. Thin film samples were precisely prepared through vacuum deposition onto cleaned fluorine-doped tin oxide (FTO)-coated glass substrates, maintained at a low pressure of 2 × 10^−6^ mbar to ensure sample integrity. During the *IP*^PE^ experiment, a negative voltage of 300 V was applied to the sample substrate, promoting electron emission from the surface. The photoelectron emission spectra were recorded using a Spectral Products^©^ 30 W deep UV deuterium light source (180–400 nm) ASBN-D130-CM, coupled with the CM110 1/8 m monochromator, which allowed precise control of the incident photon wavelength. To measure the photocurrent flowing in the circuit under illumination, a Keithley 6517B electrometer/high resistance meter was utilized, providing accurate and sensitive readings. An energy scan of the incident photons was performed by systematically changing the wavelength with the monochromator in 1 nm steps, covering the range from 280 to 180 nm. The *IP*^PE^ was subsequently estimated by identifying the intersection points of the extrapolated linear portion of the dependence of the square root of the time derivative of voltage (d*U*/d*t*)^1/2^ with respect to the incident photon energy (*h*ν) and the *h*ν axis. This approach allowed for a precise determination of the *IP*^PE^, facilitating a deeper understanding of the electron behavior and electronic properties of the studied materials.

UV–vis absorbance, photoluminescence (PL), and phosphorescence spectroscopy were used to study the optical properties of the compounds under investigation. For UV–vis absorbance spectra, solutions or films of the compounds were analyzed using an Avantes AvaSpec-2048XL spectrometer, which allowed us to assess the compounds’ absorption characteristics across the ultraviolet and visible regions. Emission spectra of solutions and films were recorded using an Edinburgh Instruments FLS980 spectrometer, enabling a detailed investigation of the emission behavior of compounds. The choice of toluene, THF, and CHCl_3_ as solvents in our study was based on their different polarities. By using these solvents, we aimed to investigate and visualize the charge transfer nature of the organic compounds under study through their spectra. To prepare the films, we used a spin-coating method by using a high-concentration THF solution (2 mg/mL). To study the phosphorescence spectra of Me-THF solutions, measurements were performed at an ultra-low temperature of 77 K, with and without a delay after excitation. This experimental setup ensured that we could capture the long-lived phosphorescence signals with high sensitivity and accuracy. To explore the temperature-dependent photoluminescence behavior of thin films, steady-state and time-resolved PL spectra were acquired using an Oxford Instruments Optistat DN2 cryostat, which was cooled with liquid nitrogen. The PL decay curves were obtained using a PicoQuant LDH-D-C-375 laser as the excitation source, operating at a wavelength of 374 nm, further allowing us to analyze the luminescence dynamics in detail. To determine photoluminescence quantum yields (PLQY), a dedicated integrated sphere with an inner diameter of 120 mm was used in conjunction with the Edinburgh Instruments FLS980 spectrometer. This specialized setup enabled accurate measurements of the PLQY, shedding light on the efficiency of light emission from the compounds. By employing this comprehensive array of spectroscopic techniques and experimental setups, we obtained a thorough understanding of the optical properties and luminescent characteristics of the studied compounds.

To accurately assess the hole and electron mobility in vacuum-deposited layers of the investigated compounds, we employed the time-of-flight (TOF) method, a reliable technique for characterizing charge transport in organic materials. Our TOF experiments involved samples with a structured configuration comprising indium–tin oxide (ITO) as the bottom electrode, a few μm thick organic layer as the active medium, and aluminum as the top electrode. The entire deposition process was carried out under a vacuum exceeding 2 × 10^−6^ mbar to ensure the integrity and purity of the layers. In our TOF setup, we utilized an EKSPLA NL300 laser, with a wavelength of 355 nm, as the excitation source to create charge carriers within the organic layer. By applying various positive and negative external voltages (*U*) to the samples using the Keithley precision 6517B electrometer, we were able to investigate hole and electron transport under different electric fields. To measure the transit time (*t*_tr_) of charge carriers, we utilized the TDS 3032C oscilloscope by Tektronix to record the photocurrent transients of holes or electrons. Subsequently, we estimated the charge mobility (μ) using the formula μ = *d*^2^/(*U* × *t*_tr_), where *d* represents the thickness of the organic layer, and *U* corresponds to the applied voltage over the sample.

Cyclic voltammetry (CV) measurements were conducted using a mAUTOLAB type III galvanostat, employing a glassy carbon working electrode in a three-electrode cell configuration. The experiments were carried out in a controlled environment with 0.1 M tetrabutylammonium hexafluorophosphate as the electrolyte and anhydrous dichloromethane as the solvent, maintaining room temperature conditions under a nitrogen atmosphere. To ensure accurate potential measurements, the system utilized silver as a quasireference electrode, while a platinum wire served as a counter electrode to facilitate the redox reactions. For calibration purposes, the potentials were standardized using the standard ferrocene/ferrocenium (*Fc*/*Fc*^+^) redox system, a well-known and widely used reference for establishing electrochemical potential scales. The CV technique provided detailed cyclic voltammograms, allowing us to analyze the redox behavior and electrochemical properties of the compounds under investigation.

## Supporting Information

File 1 Additional steady-state, time-resolved photoluminescence spectra, photoluminescence decay curves, charge transport characteristics, IR, and NMR spectra.
