# Supplementary material for: Aromatic systems with two and three pyridine-2,6-dicarbazolyl-3,5-dicarbonitrile fragments as electron-transporting organic semiconductors exhibiting long-lived emissions
Source: Beilstein J Org Chem. 2023 Dec 12;19:1867–80. doi: 10.3762/bjoc.19.139 (PMC10729123; doi:10.3762/bjoc.19.139)
Supplement: File 1 — Additional steady-state, time-resolved photoluminescence spectra, photoluminescence decay curves, charge transport characteristics, IR, and NMR spectra. [file Beilstein_J_Org_Chem-19-1867-s001.pdf]

BEILSTEIN JOURNAL OF ORGANIC CHEMISTRY

Supporting Information

for

Aromatic systems with two and three pyridine-2,6-dicarbazolyl-3,5-dicarbonitrile fragments as electron-transporting organic semiconductors exhibiting long-lived emissions

Karolis Leitonas, Brigita Vigante, Dmytro Volyniuk, Audrius Bucinskas, Pavels Dimitrijevs, Sindija Lapcinska, Pavel Arsenyan and Juozas Vidas Grazulevicius

Beilstein J. Org. Chem. **2023,** *19,* 1867–1880. doi:10.3762/bjoc.19.139

Additional steady-state, time-resolved photoluminescence spectra, photoluminescence decay curves, charge transport characteristics, IR, and NMR spectra

License and Terms: This is a supporting information file under the terms of the Creative Commons Attribution License (https://creativecommons.org/licenses/by/4.0). Please note that the reuse, redistribution and reproduction in particular requires that the author(s) and source are credited and that individual graphics may be subject to special legal provisions.
The license is subject to the *Beilstein Journal of Organic Chemistry* terms and conditions: (https://www.beilstein-journals.org/bjoc/terms)

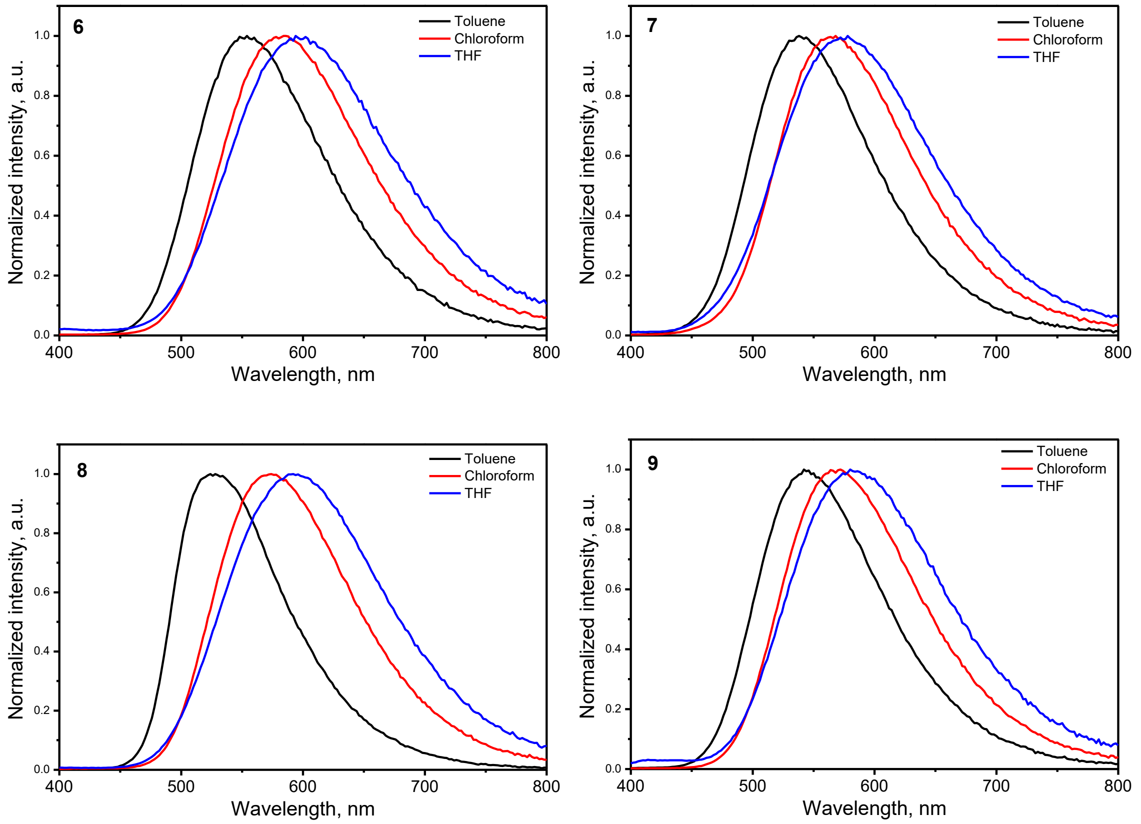

Figure S1: PL spectra of the dilute toluene, THF and chloroform solutions (10^{-5} M) of compounds **6**–**9**.

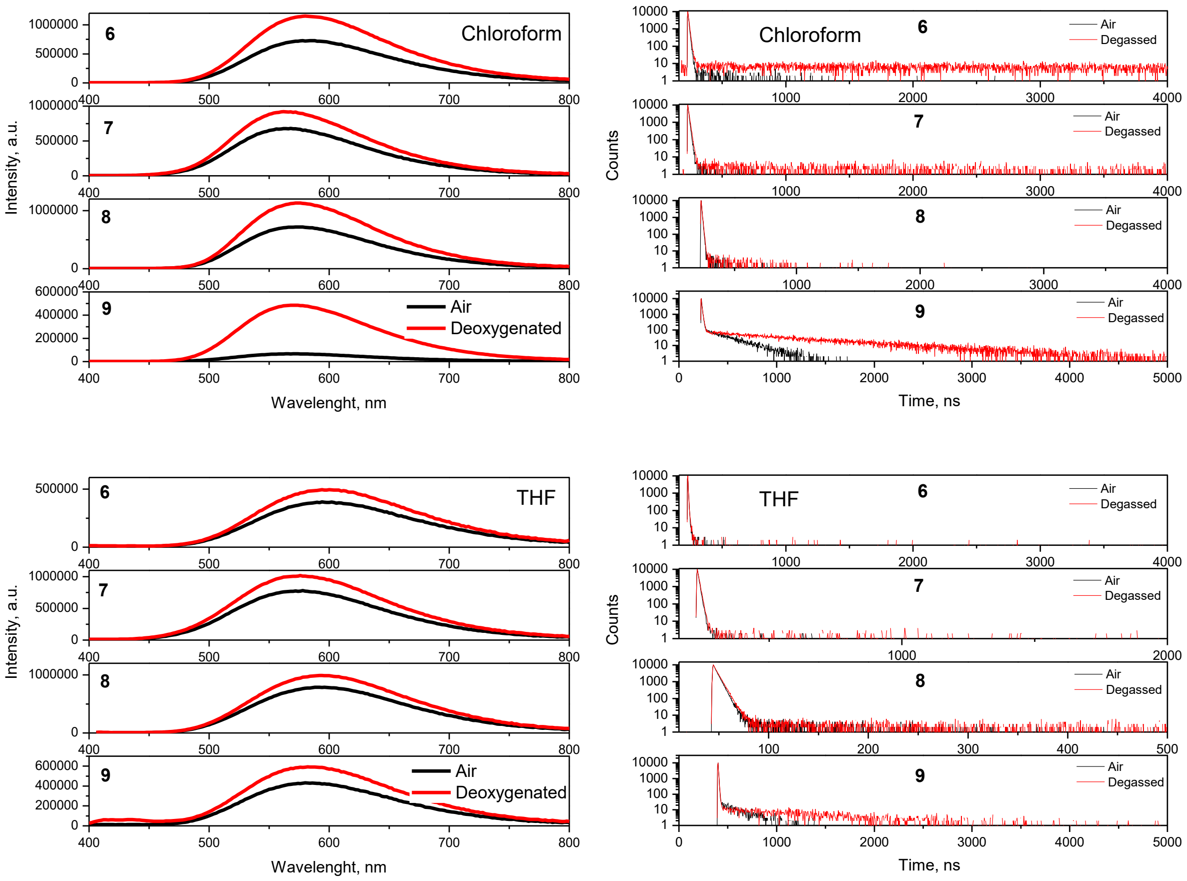

a) b)

Figure S2: PL intensities (a) and PL decays (b) of non-deoxygenated (as prepared) and deoxygenated chloroform and THF solutions of compounds **6**–**9**.

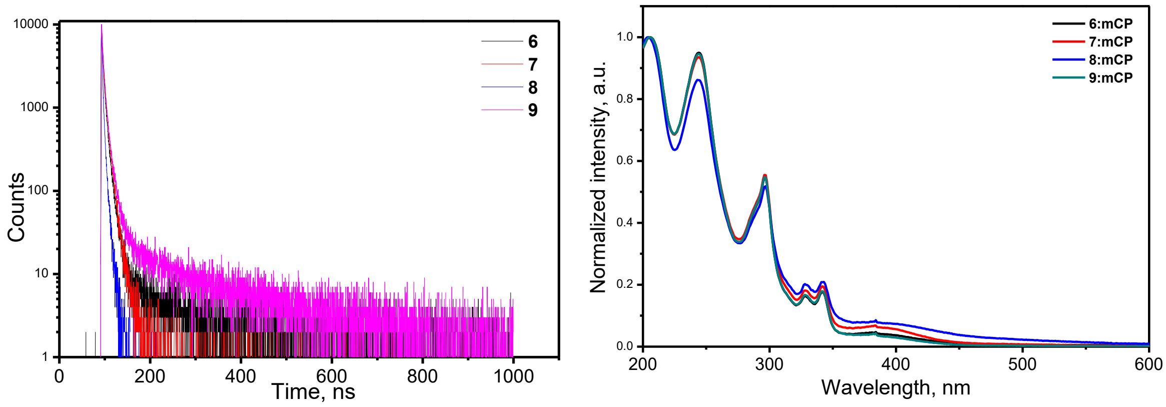

Figure S3: PL decay transients of neat films of compounds **6**–**9** and absorption spectra of mCP host-based films of compounds **6**–**9** (10 wt %).

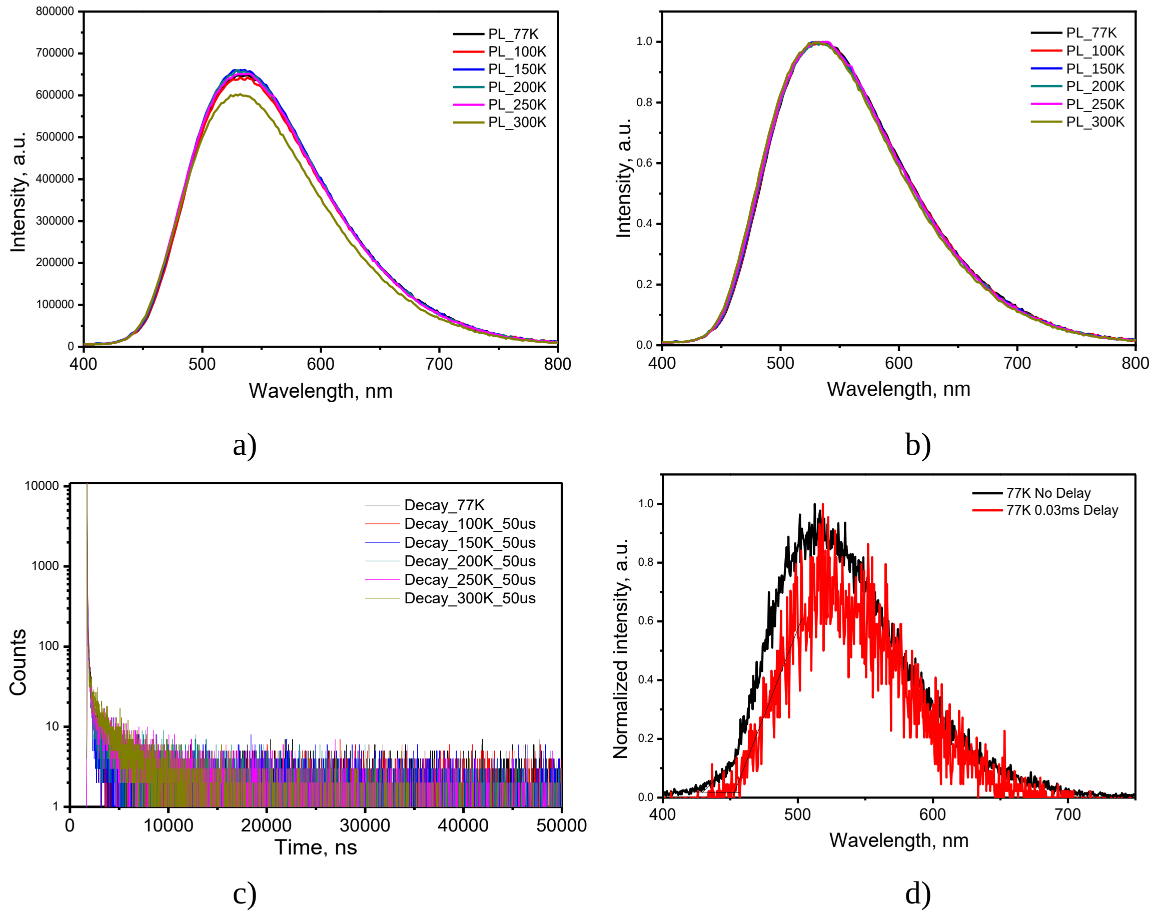

Figure S4: Non-normalized (a) and normalized (b) PL spectra and PL decay transients (c) of the film of compound **6** (10 wt %) dispersed in mCP recorded at different temperatures. PL and phosphorescence spectra of the same sample recorded 77K (d). Phosphorescence was separated from fluorescence using a delay of 0.03 ms after excitation. Excitation wavelengths were 350 and 374 nm, respectively, for recording PL spectra and PL decay transients.

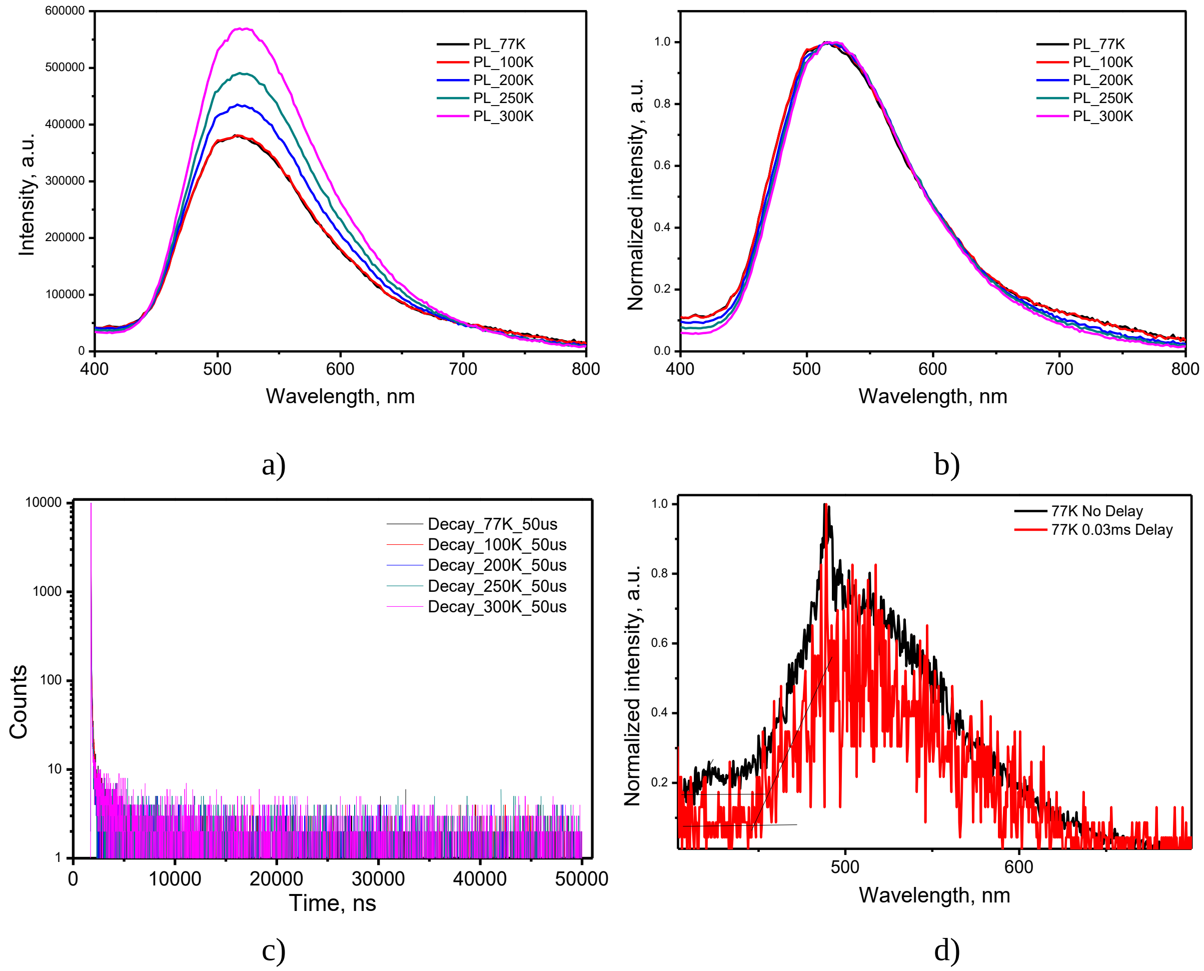

Figure S5: Non-normalized (a) and normalized (b) PL spectra and PL decay transients (c) of the film of compound **7** (10 wt %) dispersed in mCP recorded at different temperatures. PL and phosphorescence spectra of the same sample recorded 77K (d). Phosphorescence was separated from fluorescence using a delay of 0.03 ms after excitation. Excitation wavelengths were 350 and 374 nm, respectively, for recording PL spectra and PL decay transients.

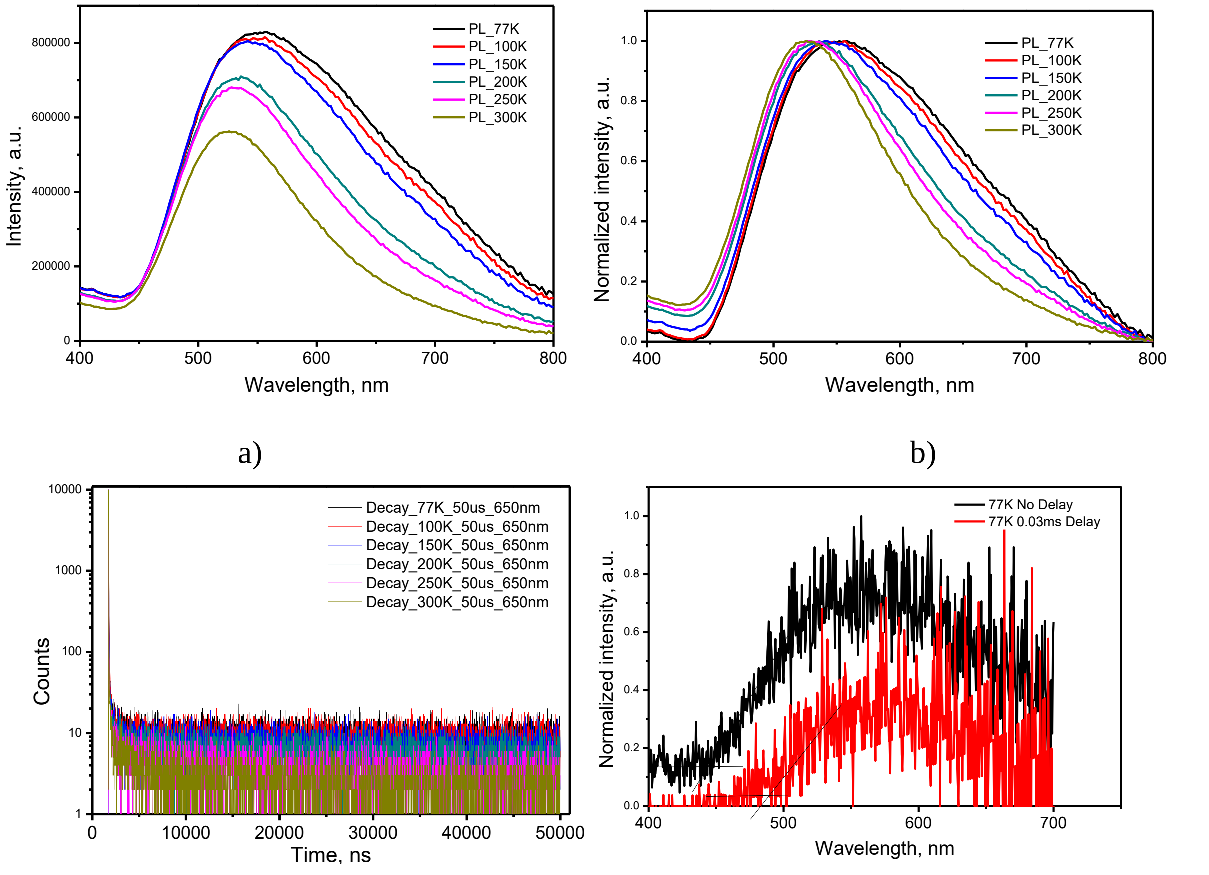

a) b)

c) d)

Figure S6: Non-normalized (a) and normalized (b) PL spectra and PL decay transients (c) of the film of compound **8** (10 wt %) dispersed in mCP recorded at different temperatures. PL and phosphorescence spectra of the same sample recorded 77K (d). Phosphorescence was separated from fluorescence using a delay of 0.03 ms after excitation. Excitation wavelengths were 350 and 374 nm, respectively, for recording PL spectra and PL decay transients.

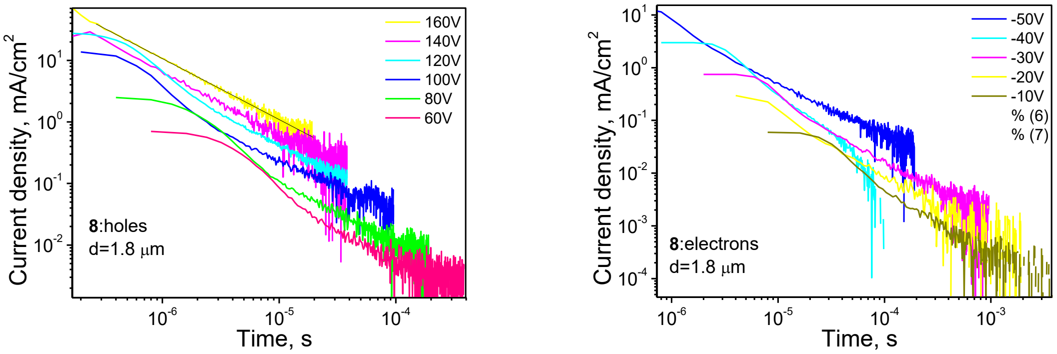

Figure S7: Examples of current transients for holes and electrons in films of compound **8** without detectable transit times.

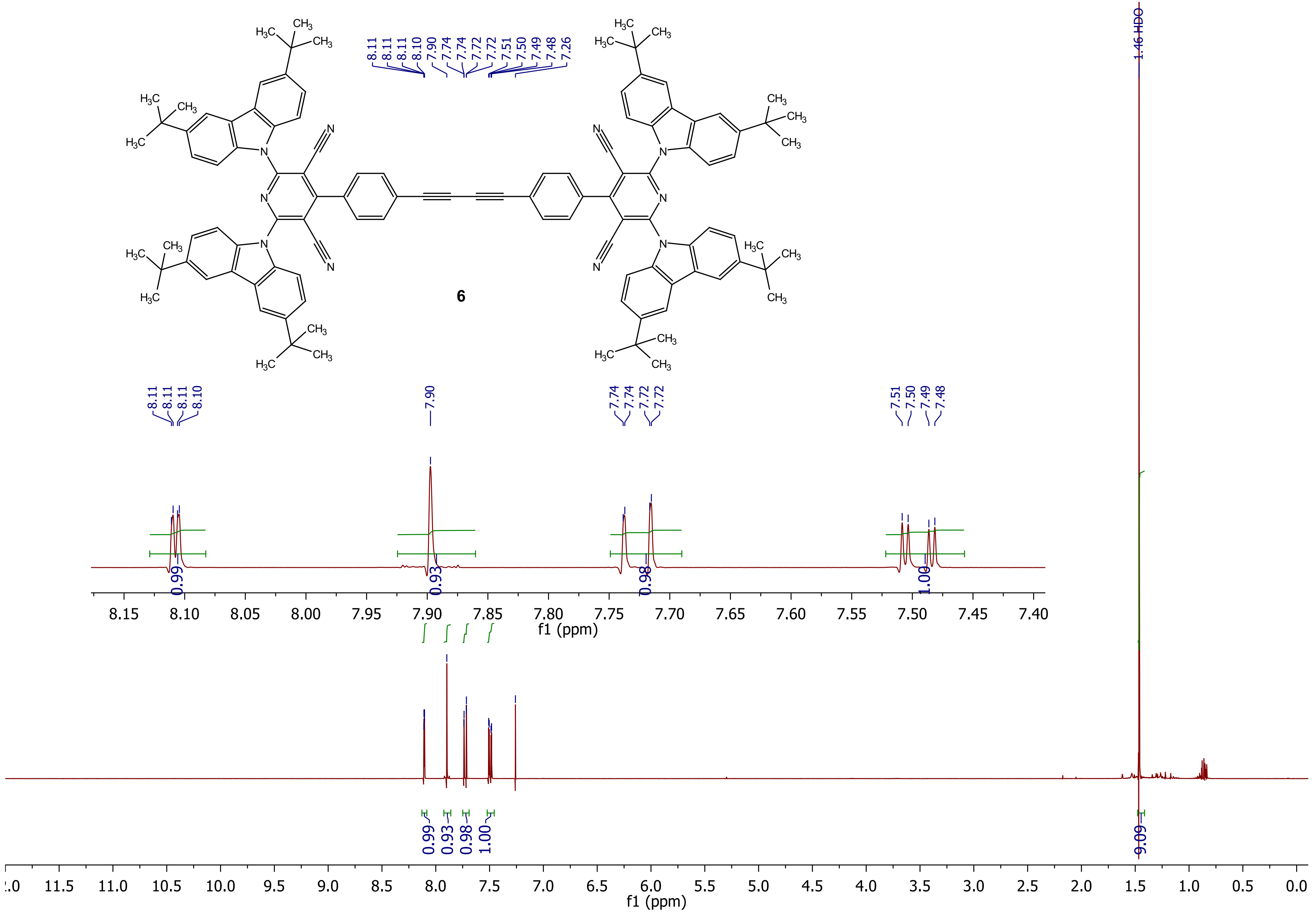
6
8.11 8.11 8.11 8.10 7.90 7.74 7.74 7.72 7.72 7.51 7.50 7.49 7.48 7.26
1.46 HDO
0.99
0.93
0.98
1.00
9.09
f1 (ppm)

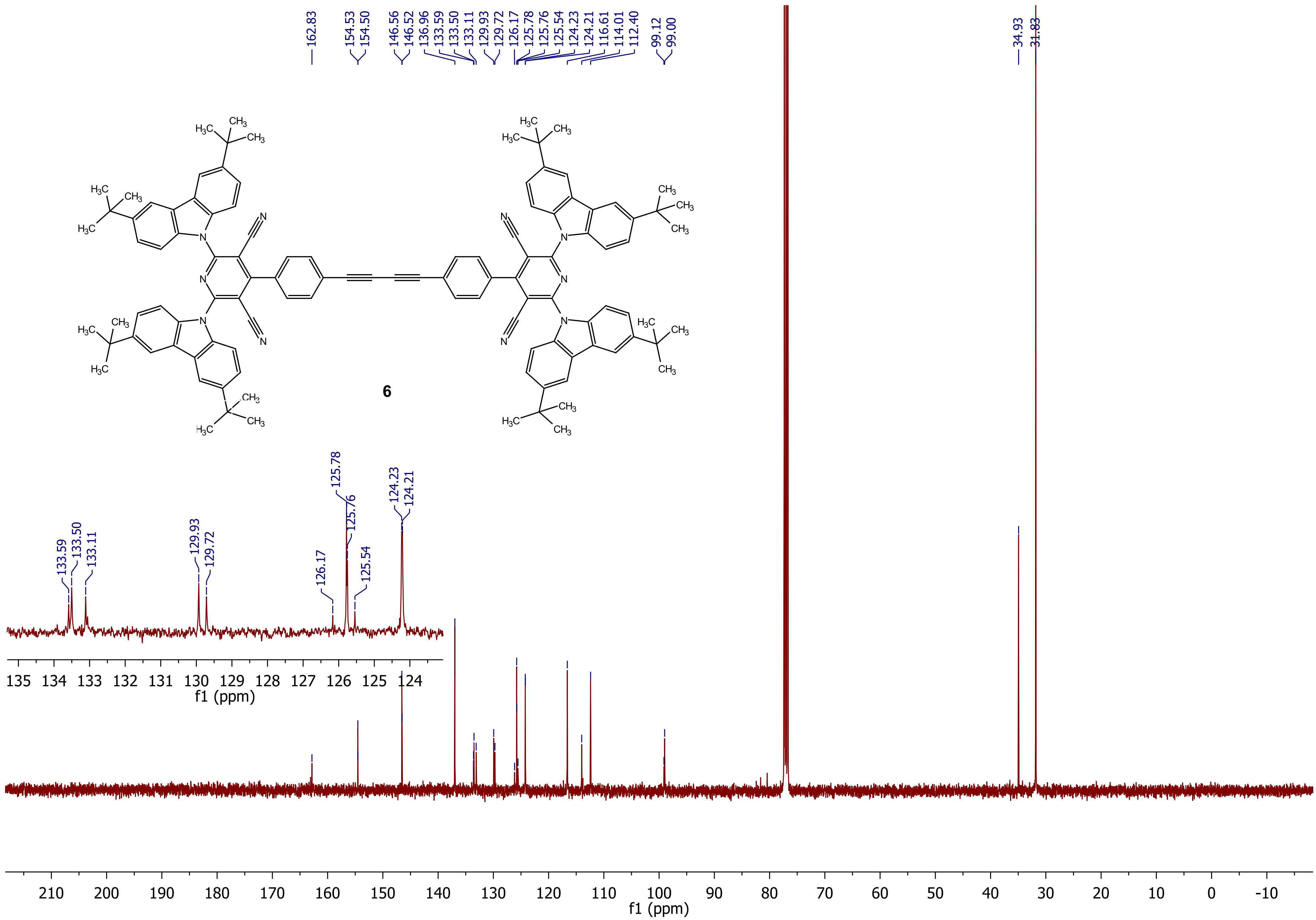
162.83
154.53
154.50
146.56
146.52
136.96
133.59
133.50
133.11
129.93
129.72
126.17
125.78
125.76
125.54
124.23
124.21
116.61
114.01
112.40
99.12
99.00
34.93
31.83
6
135 134 133 132 131 130 129 128 127 126 125 124
f1 (ppm)
210 200 190 180 170 160 150 140 130 120 110 100 90 80 70 60 50 40 30 20 10 0 -10
f1 (ppm)

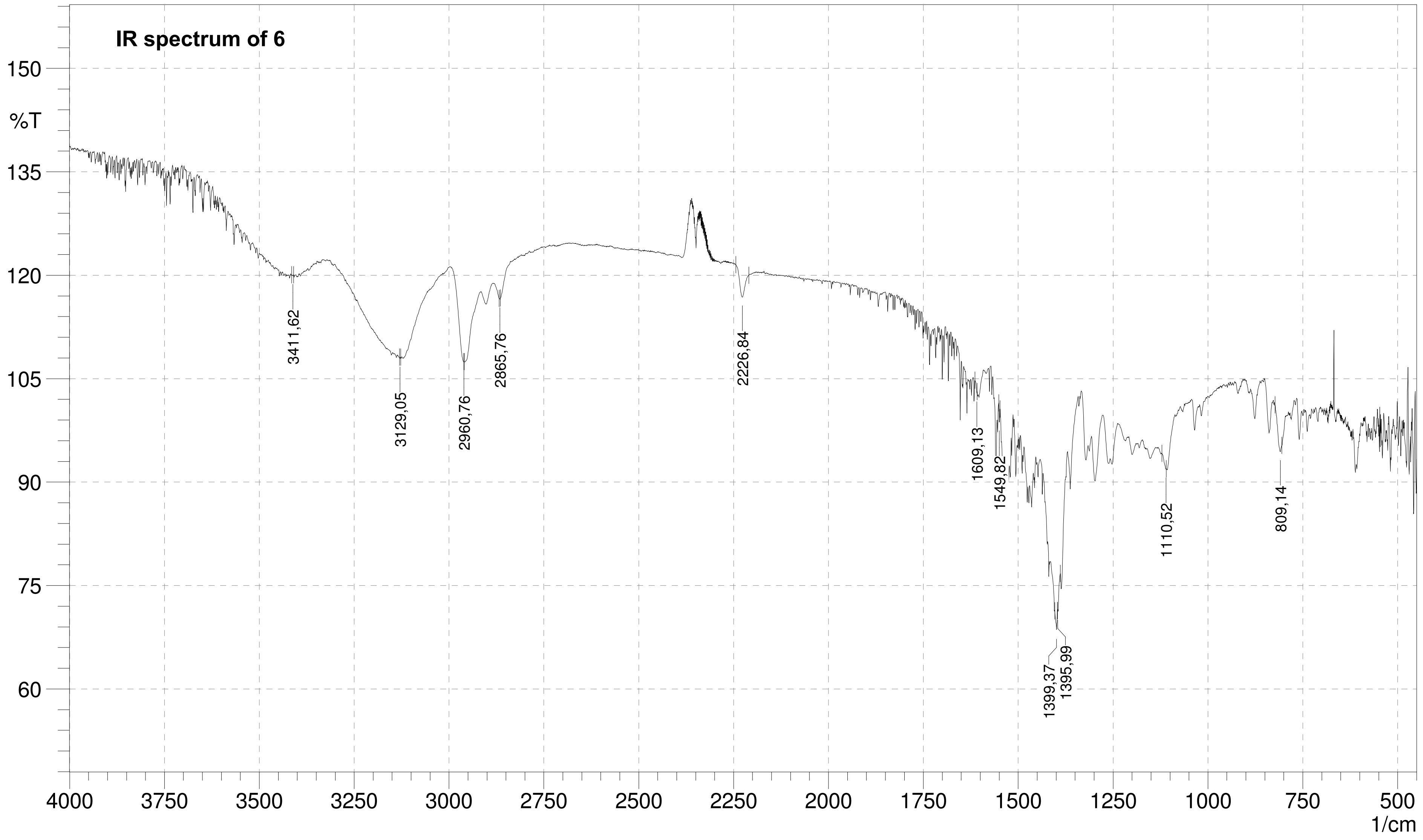
IR spectrum of 6
%T
150
135
120
105
90
75
60
3411,62
3129,05
2960,76
2865,76
2226,84
1609,13
1549,82
1399,37
1395,99
1110,52
809,14
4000
3750
3500
3250
3000
2750
2500
2250
2000
1750
1500
1250
1000
750
500
1/cm

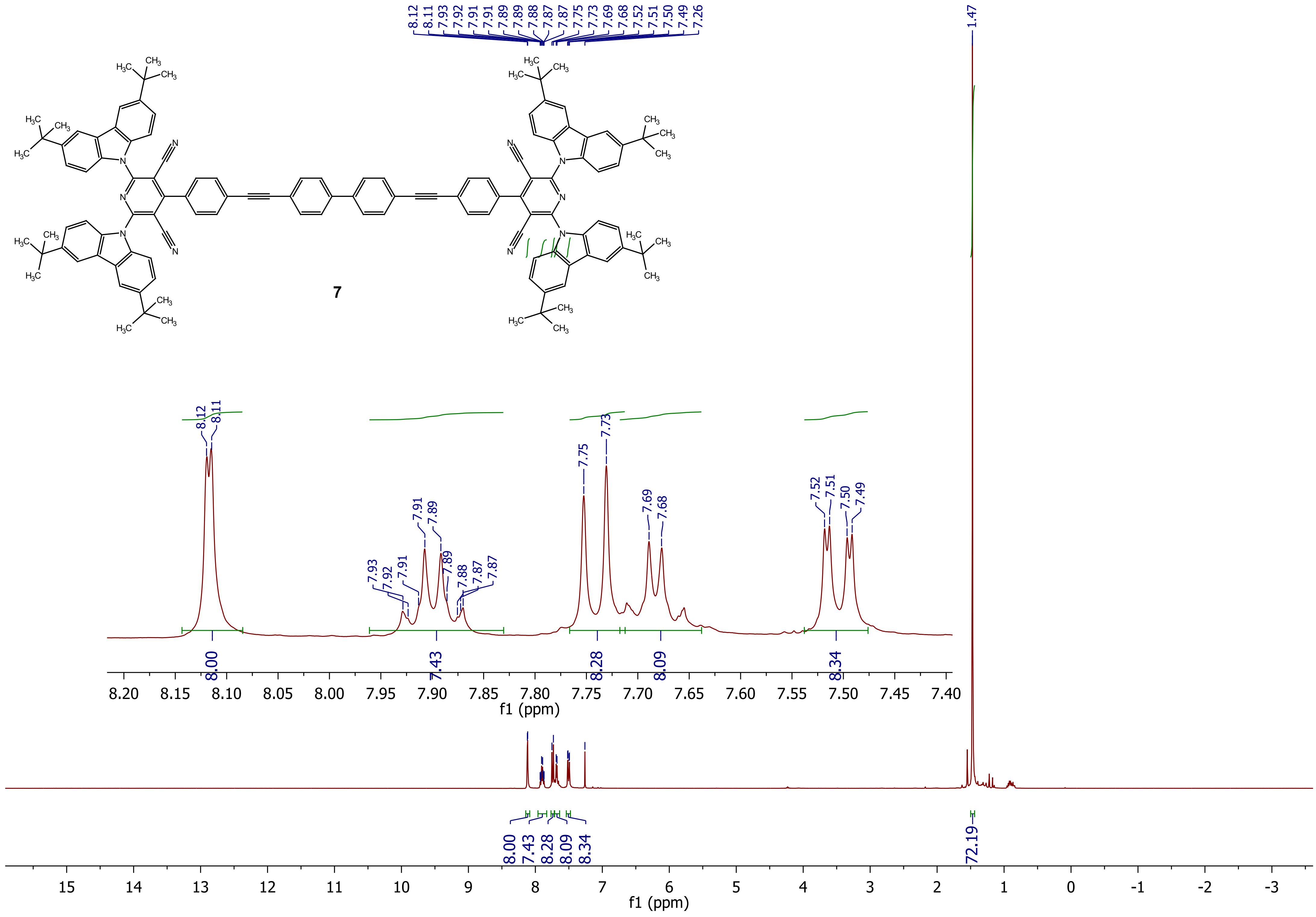
8.12 8.11 7.93 7.92 7.91 7.91 7.89 7.89 7.88 7.87 7.87 7.75 7.73 7.69 7.68 7.52 7.51 7.50 7.49 7.26
1.47
7
8.00
7.43
8.28
8.09
8.34
72.19
f1 (ppm)
f1 (ppm)

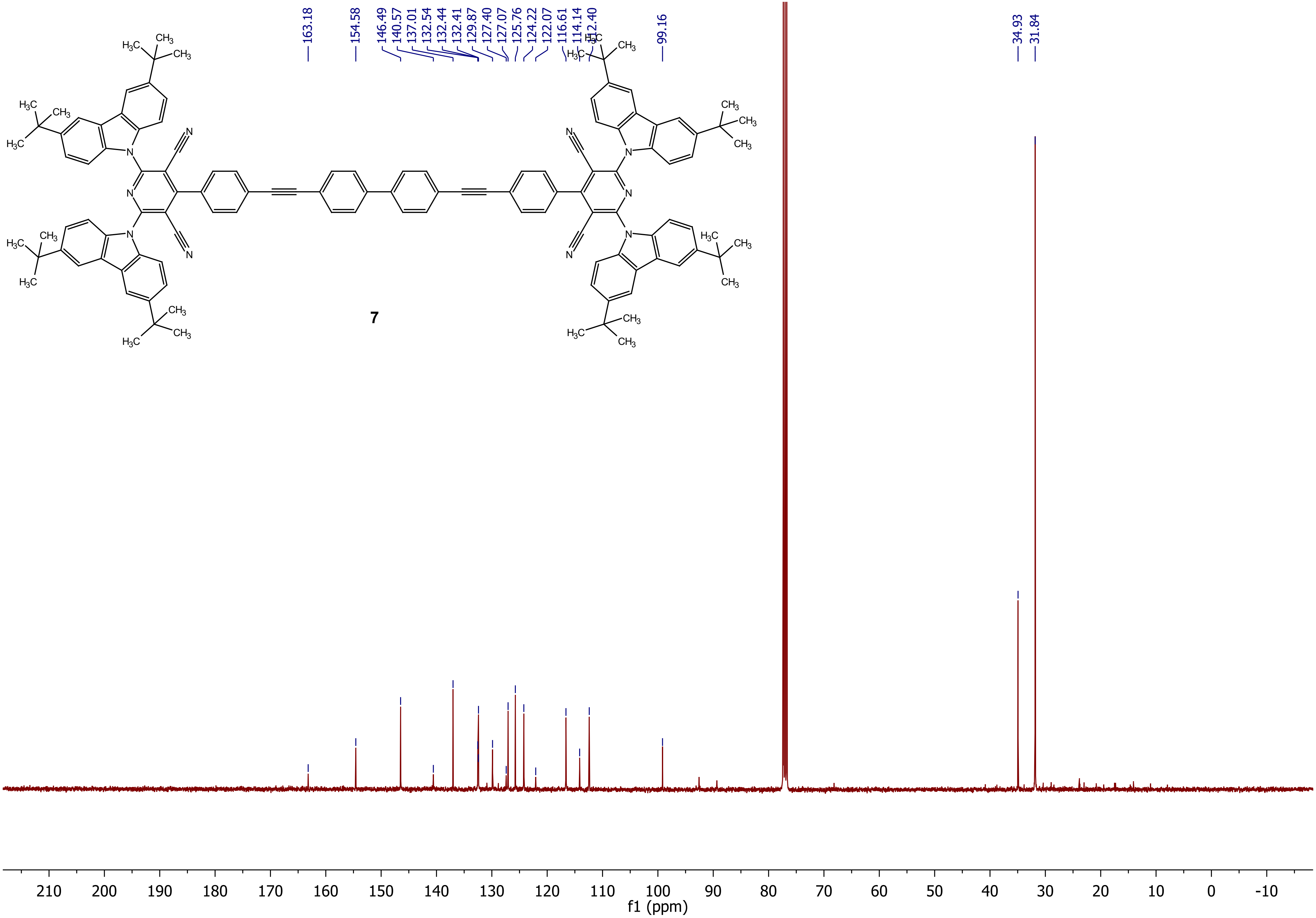
163.18
154.58
146.49
140.57
137.01
132.54
132.44
132.41
129.87
127.40
127.07
125.76
124.22
122.07
116.61
114.14
112.40
99.16
34.93
31.84
7
210 200 190 180 170 160 150 140 130 120 110 100 90 80 70 60 50 40 30 20 10 0 -10
f1 (ppm)

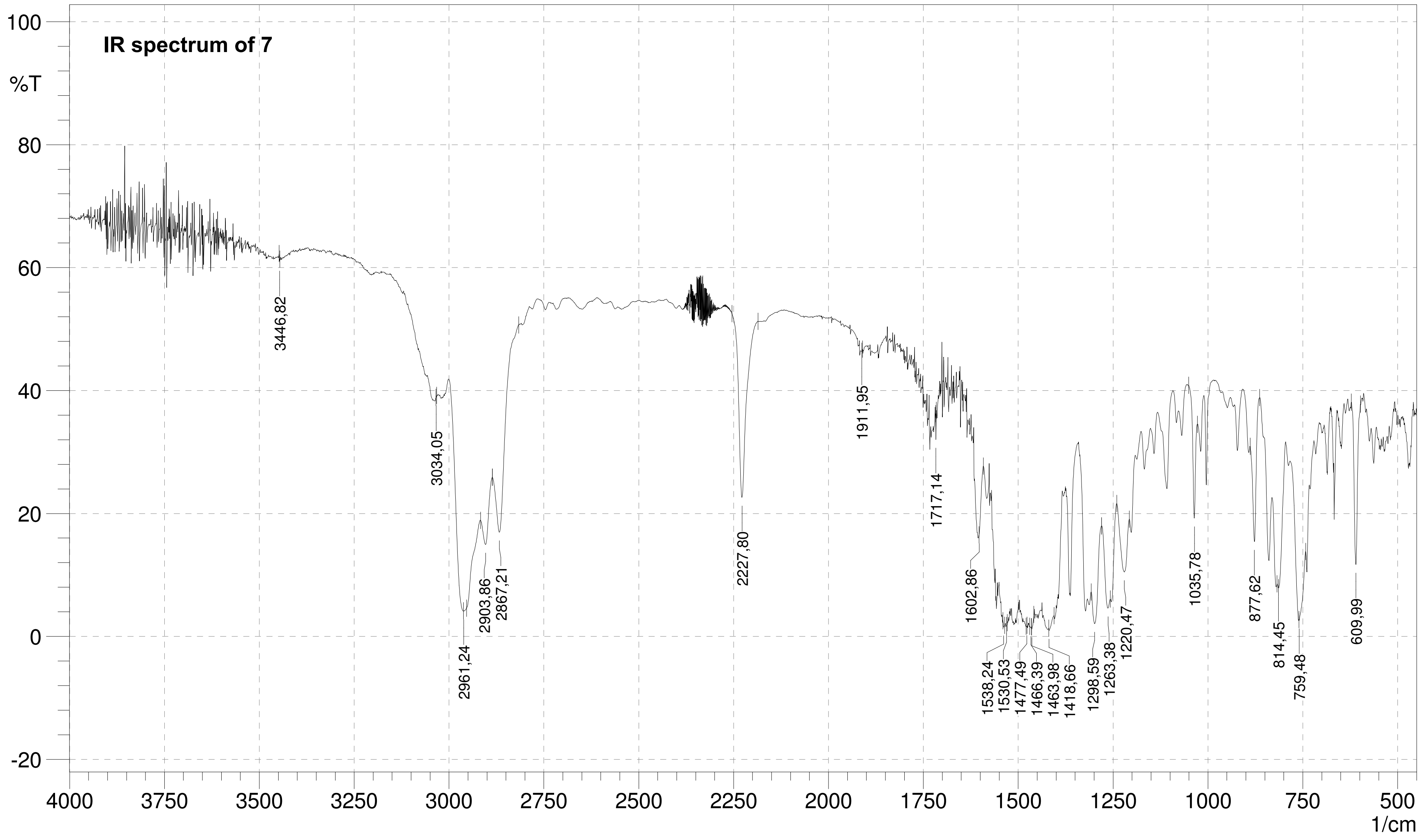
IR spectrum of 7
%T
100
80
60
40
20
0
-20
3446,82
3034,05
2961,24
2903,86
2867,21
2227,80
1911,95
1717,14
1602,86
1538,24
1530,53
1477,49
1466,39
1463,98
1418,66
1298,59
1263,38
1220,47
1035,78
877,62
814,45
759,48
609,99
4000
3750
3500
3250
3000
2750
2500
2250
2000
1750
1500
1250
1000
750
500
1/cm

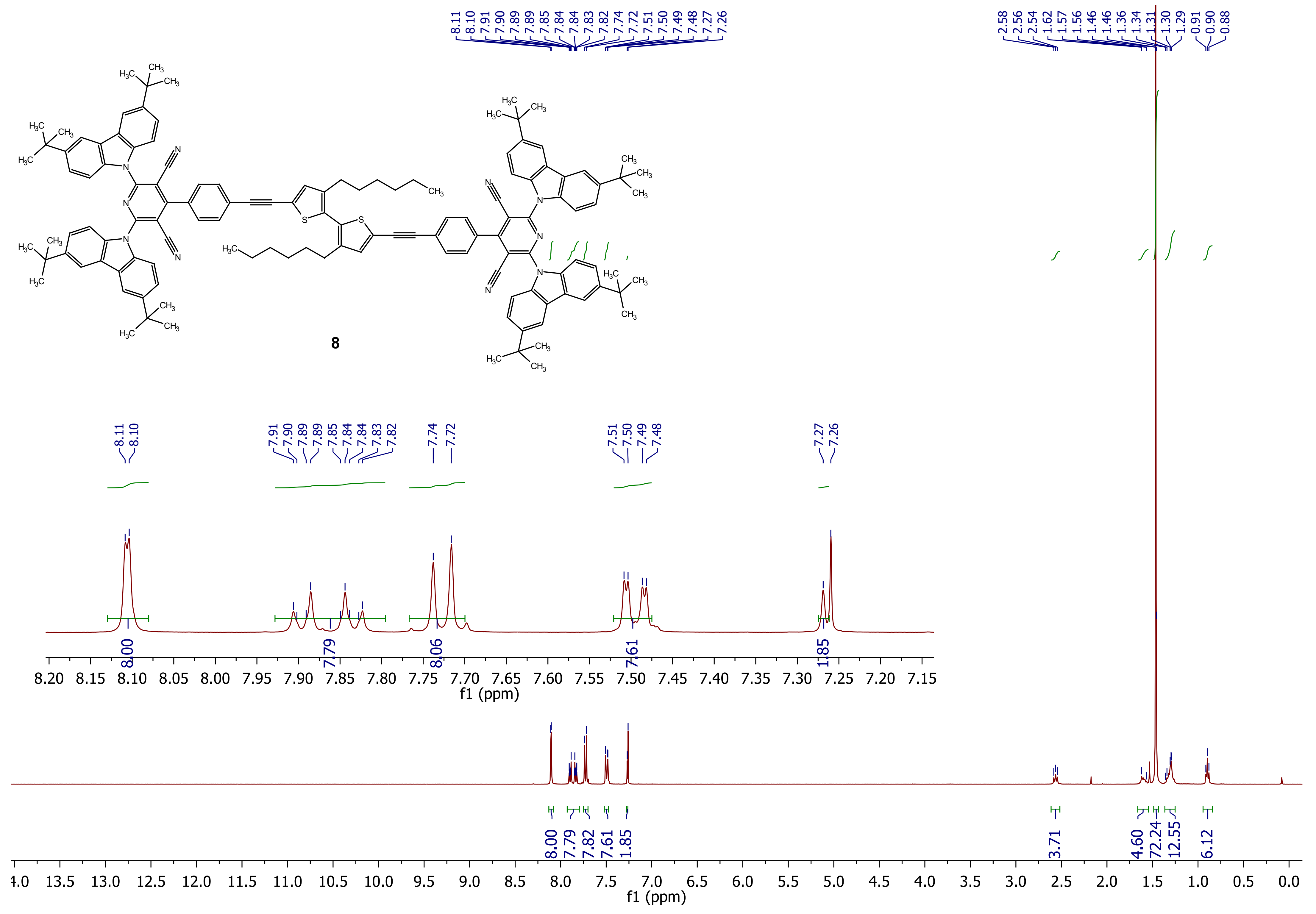
8
8.00
7.79
8.06
7.61
1.85
8.00
7.79
7.82
7.61
1.85
3.71
4.60
72.24
12.55
6.12
f1 (ppm)
f1 (ppm)

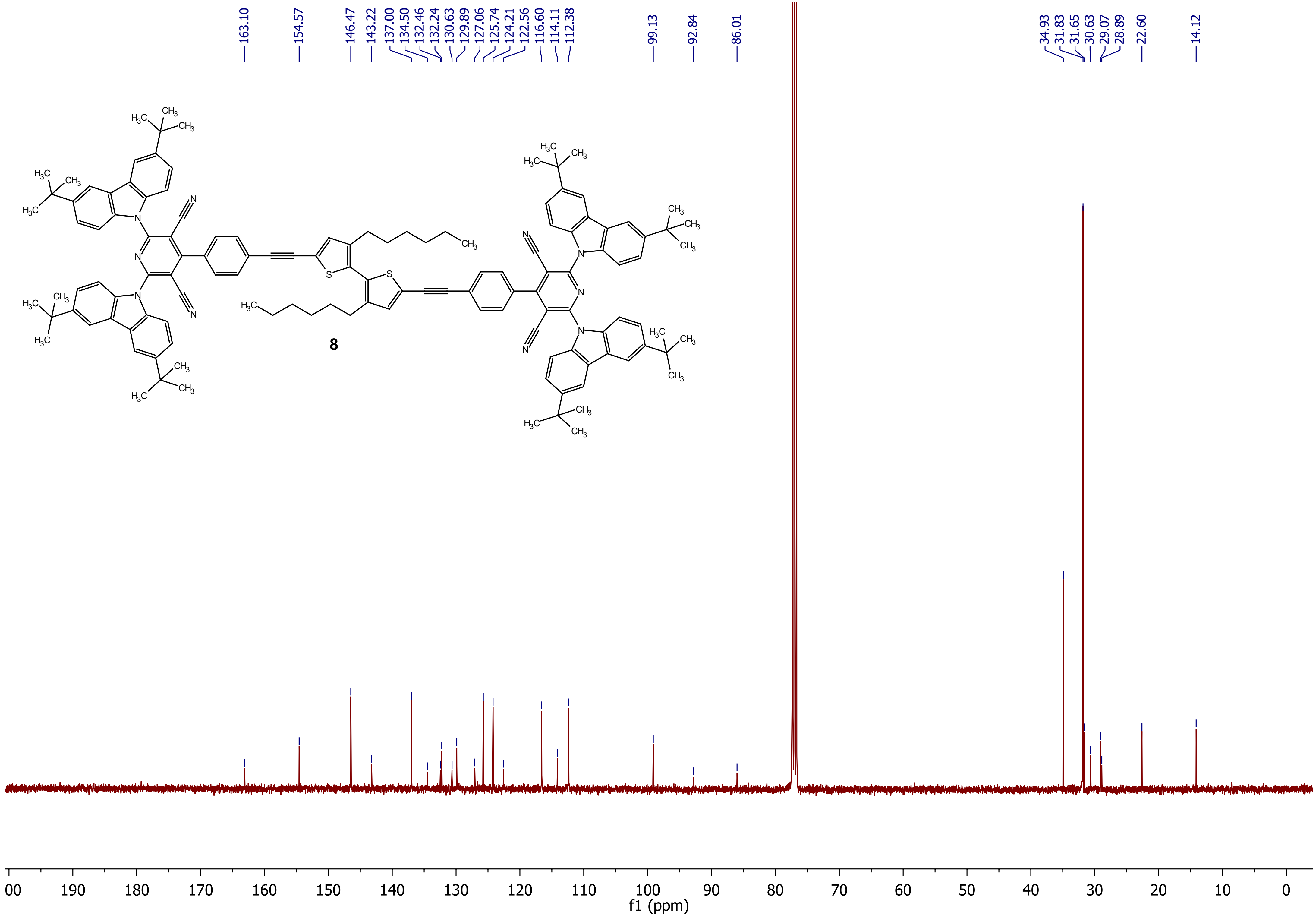
163.10
154.57
146.47
143.22
137.00
134.50
132.46
132.24
130.63
129.89
127.06
125.74
124.21
122.56
116.60
114.11
112.38
99.13
92.84
86.01
34.93
31.83
31.65
30.63
29.07
28.89
22.60
14.12
8
f1 (ppm)

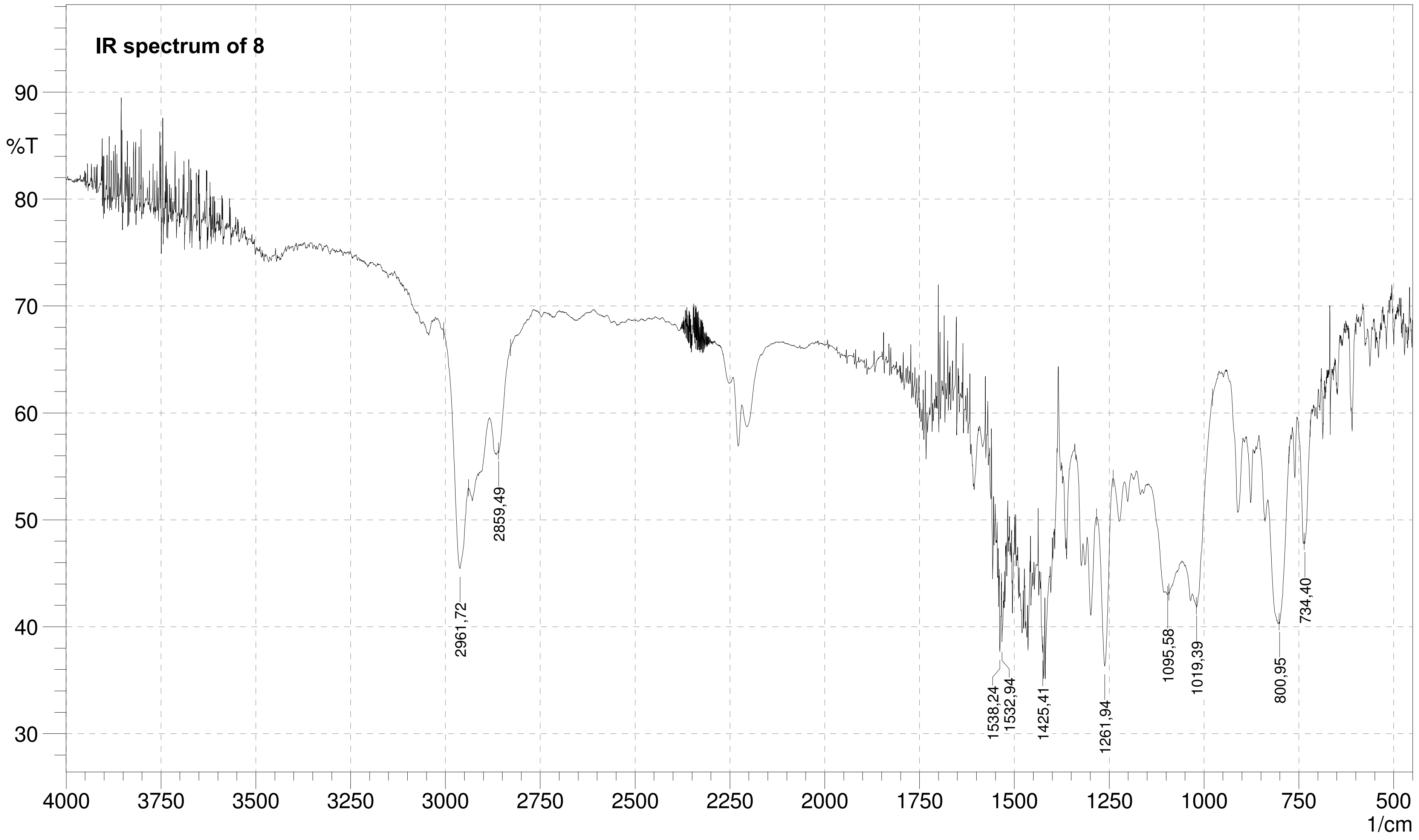

IR spectrum of 8
%T
90
80
70
60
50
40
30
4000
3750
3500
3250
3000
2750
2500
2250
2000
1750
1500
1250
1000
750
500
1/cm
2961,72
2859,49
1538,24
1532,94
1425,41
1261,94
1095,58
1019,39
800,95
734,40

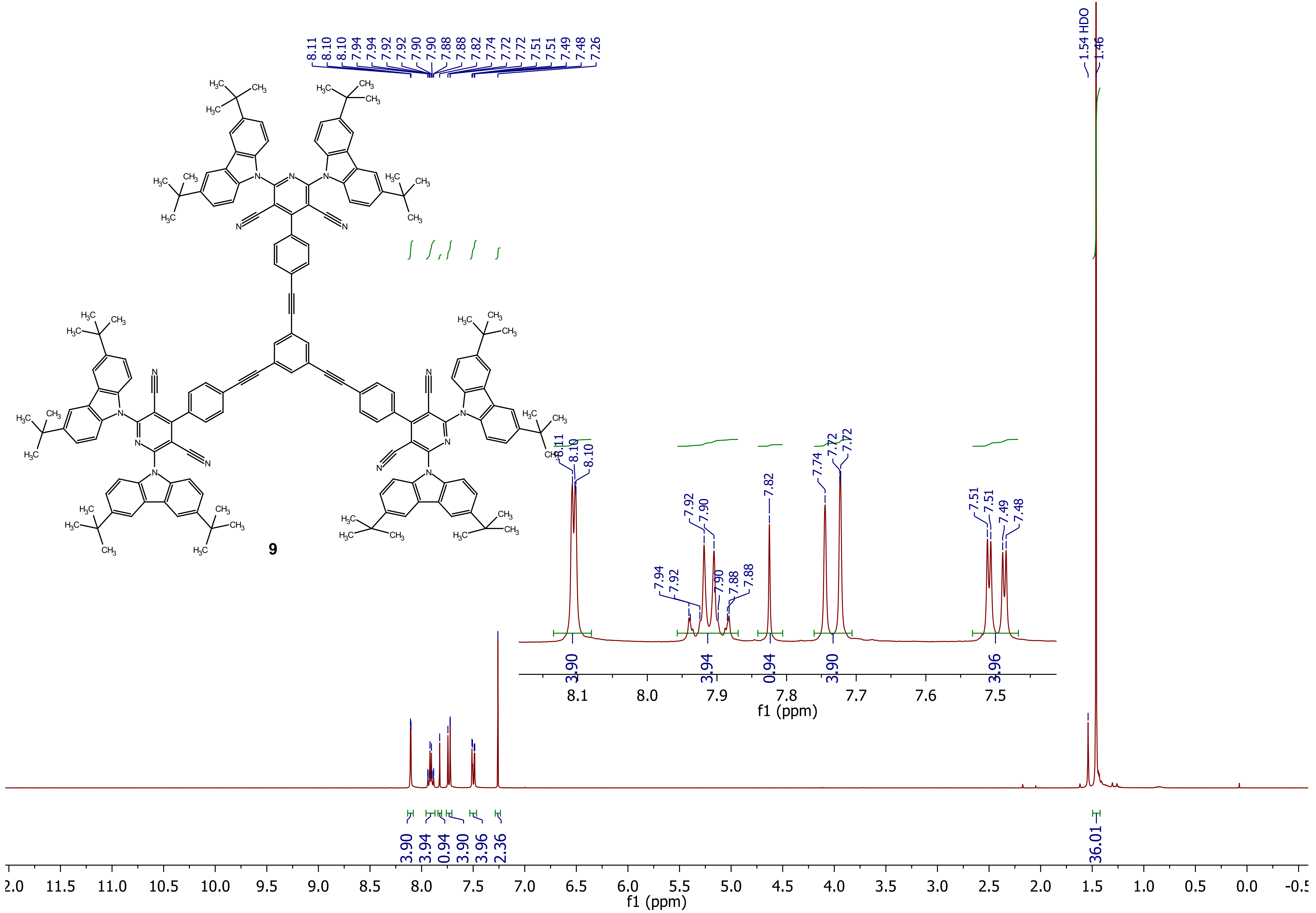

8.11 8.10 8.10 7.94 7.92 7.92 7.90 7.90 7.88 7.88 7.82 7.74 7.72 7.72 7.51 7.51 7.49 7.48 7.26
1.54 HDO
1.46
9
3.90
3.94
0.94
3.90
3.96
2.36
36.01
f1 (ppm)

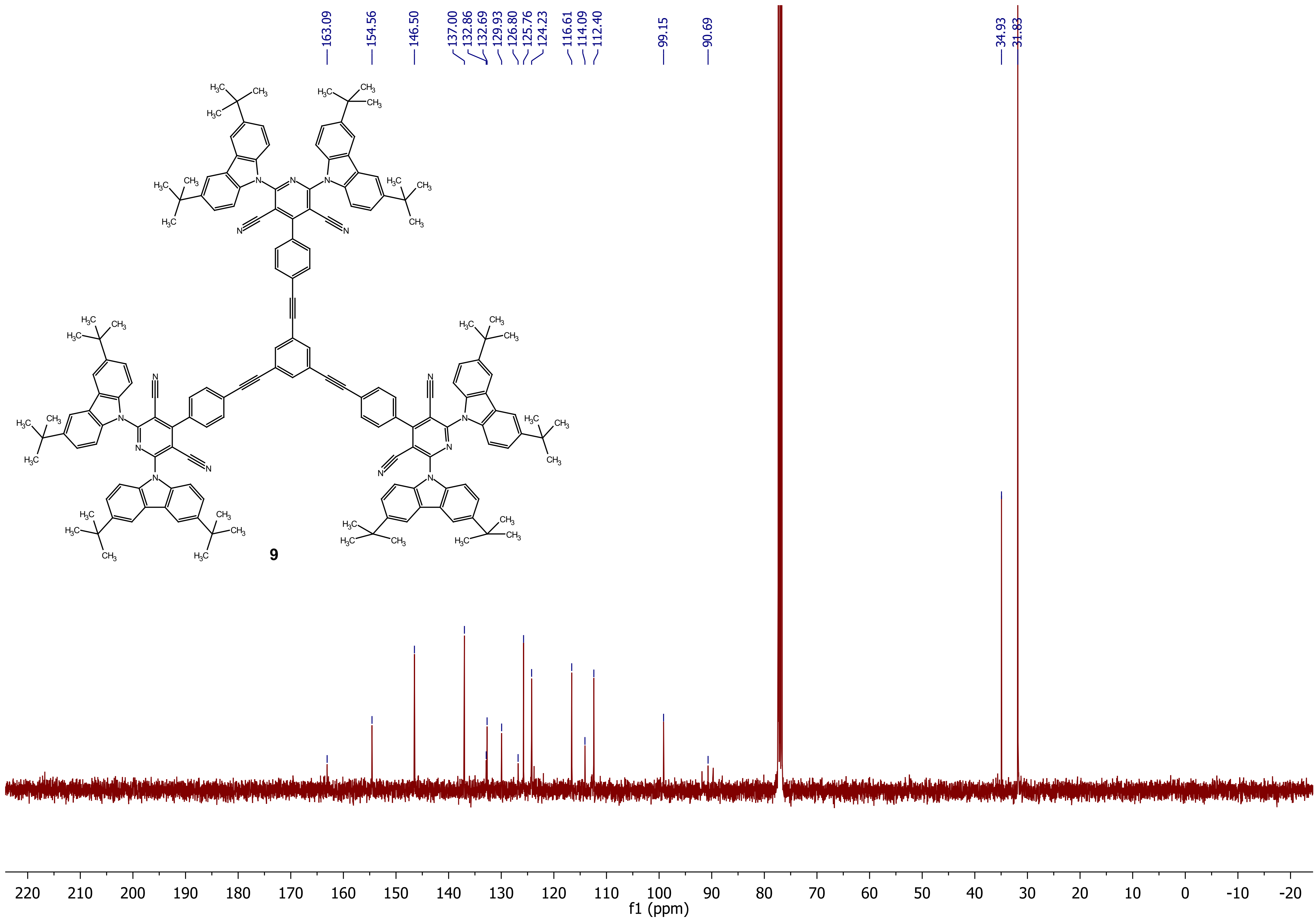

163.09
154.56
146.50
137.00
132.86
132.69
129.93
126.80
125.76
124.23
116.61
114.09
112.40
99.15
90.69
34.93
31.83
9
220
210
200
190
180
170
160
150
140
130
120
110
100
90
80
70
60
50
40
30
20
10
0
-10
-20
f1 (ppm)

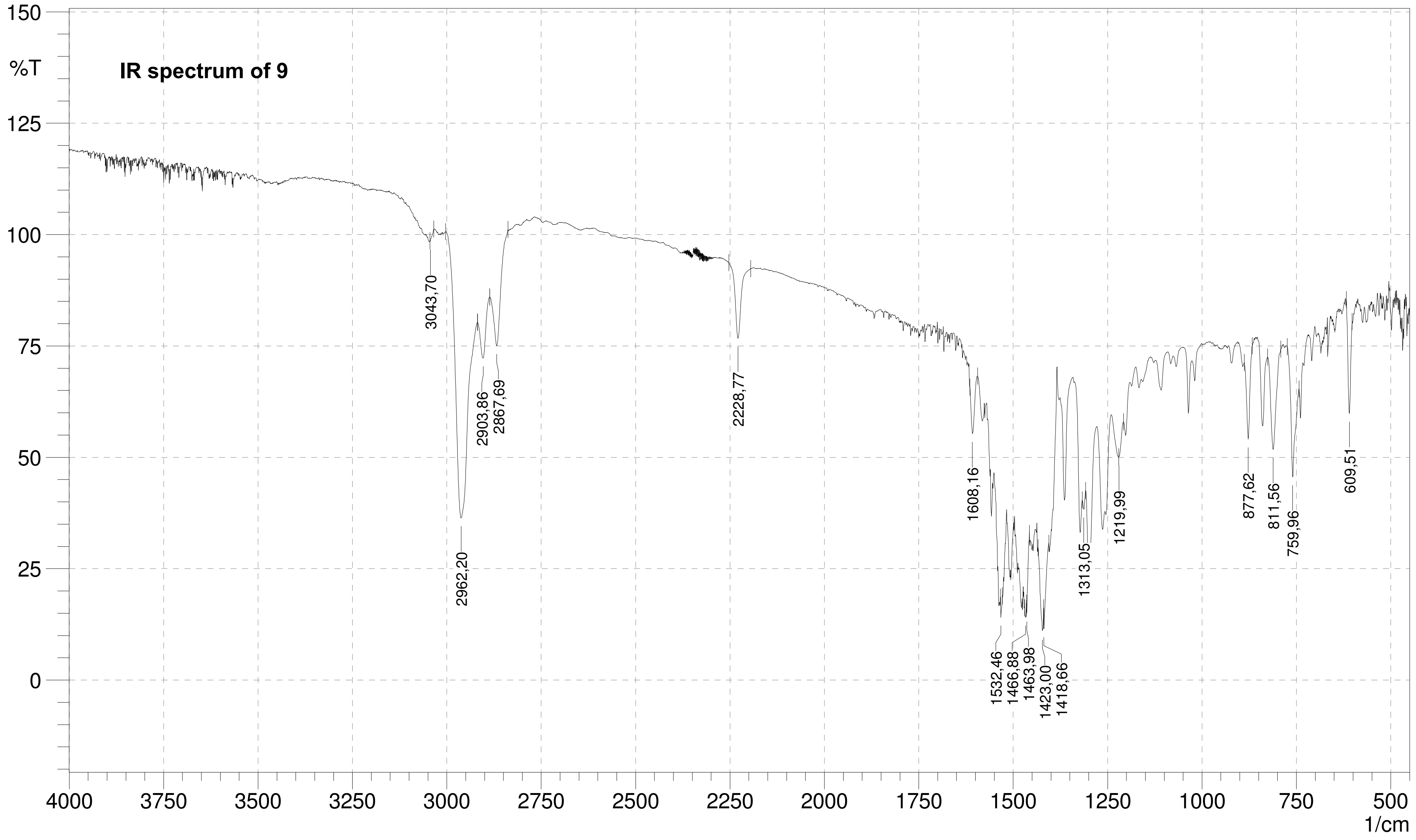
IR spectrum of 9
%T
150
125
100
75
50
25
0
3043,70
2962,20
2903,86
2867,69
2228,77
1608,16
1532,46
1466,88
1463,98
1423,00
1418,66
1313,05
1219,99
877,62
811,56
759,96
609,51
4000
3750
3500
3250
3000
2750
2500
2250
2000
1750
1500
1250
1000
750
500
1/cm
